# ALDOA‐Mediated Metabolic Reprogramming is a Targetable Vulnerability for Ferroptosis Sensitization in Cancer

**DOI:** 10.1002/advs.202511880

**Published:** 2025-11-11

**Authors:** Pengqi Wang, Kezhang He, Bowen Wang, Wei Zhou, Ying Zhang, Tianhua Ma, Sheng Ding

**Affiliations:** ^1^ New Cornerstone Science Laboratory, School of Pharmaceutical Sciences Tsinghua University Beijing 100084 China; ^2^ School of Life Sciences Tsinghua University Beijing 100084 China; ^3^ Tsinghua‐Peking Joint Center for Life Sciences Tsinghua University Beijing 100084 China

**Keywords:** ALDOA, autophagy, cancer, ferroptosis, lipid metabolism, metabolic reprogramming

## Abstract

Ferroptosis presents great potential for cancer therapy, either alone or in combination with classical therapy. However, inducing ferroptosis by targeting canonical ferroptosis suppressors that directly inhibit lipid peroxidation non‐selectively induces ferroptosis in both cancerous and normal cells, thereby limiting its therapeutic potential. In this study, it is revealed that aldolase A (ALDOA) reprograms lipid metabolism to resist ferroptosis in cancer cells and identifies ALDOA as a targetable vulnerability for ferroptosis sensitization. Cancer cells with ALDOA suppression exhibit increased susceptibility to ferroptosis—a response less obvious in normal cells. Mechanistically, ALDOA depletion induces significant accumulation of fructose 1,6‐bisphosphate in cancer cells, thereby enhancing autophagy‐dependent degradation of phospholipid‐modifying enzymes. These alterations increase the ratio of phospholipids containing pro‐ferroptotic polyunsaturated fatty acids over anti‐ferroptotic monounsaturated fatty acids, culminating in heightened ferroptosis sensitivity. Moreover, ALDOA inhibitors selectively promote ferroptosis in cancer cells, both in vitro and in vivo. Collectively, the findings reveal that ALDOA‐mediated metabolic reprogramming is a targetable vulnerability for ferroptosis sensitization in cancer.

## Graphical abstract

ALDOA‐depletion‐mediated glycolytic reprogramming promotes autophagy‐mediated degradation of MBOAT2, increasing the ratio of phospholipids containing pro‐ferroptotic polyunsaturated fatty acids over anti‐ferroptotic monounsaturated fatty acids, and thus sensitizing cancer cells to ferroptosis. Targeting this metabolic cascade reveals a cancer‐specific vulnerability that can be exploited for safe and effective ferroptosis‐based therapy.

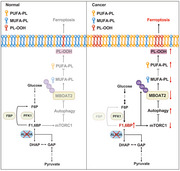

## 1 Introduction

Ferroptosis, a form of regulated cell death driven by the accumulation of toxic oxidized lipids, has been implicated across a wide range of diseases,^[^ ^1^ ^]^ including cancer,^[^ ^2^, ^3^, ^4^ ^]^ neurodegenerative disorders,^[^ ^5^, ^6^ ^]^ as well as organ ischemia‐reperfusion injuries.^[^ ^7^, ^8^ ^]^ Notably, ferroptosis induction has shown great potential in cancer therapy by overcoming resistance to conventional therapies and complementing established modalities such as chemotherapy, immunotherapy, and radiotherapy.^[^ ^3^, ^4^, ^9^, ^10^, ^11^, ^12^ ^]^ However, concerns about its potential side effects have been noted.^[^ ^12^, ^13^ ^]^ In fact, recent research has shown that various normal tissues are also susceptible to ferroptosis,^[^ ^14^ ^]^ impeding the application of ferroptosis induction as a viable cancer therapeutic strategy. Indeed, the downregulation or inhibition of known ferroptosis suppressors, such as glutathione peroxidase 4 (GPX4),^[^ ^15^ ^]^ ferroptosis suppressor protein 1 (FSP1),^[^ ^16^, ^17^ ^]^ dihydroorotate dehydrogenase (DHODH),^[^ ^18^ ^]^ and 7‐dehydrocholesterol (7‐DHC) synthesis proteins,^[^ ^19^, ^20^ ^]^ promotes ferroptosis non‐selectively in both cancerous and normal cells. This underscores the urgent need to identify ferroptosis vulnerabilities in cancer cells to overcome these limitations and enable effective cancer therapies through ferroptosis induction.

As ferroptosis has been recognized as a metabolism‐linked cell death,^[^ ^21^ ^]^ distinct metabolic reprogramming in cancer cells offers a promising opportunity to identify cancer‐specific ferroptosis regulators. Aerobic glycolysis, also known as the Warburg effect, is one of the hallmarks of cancer cells.^[^ ^22^ ^]^ This metabolic reprogramming not only fuels macromolecule biosynthesis for rapid cell proliferation but also mitigates oxidative stress to enhance cancer survival.^[^ ^23^ ^]^ Aldolase, a key glycolytic enzyme, is aberrantly expressed across a broad spectrum of cancers and plays crucial roles in tumor maintenance.^[^ ^24^, ^25^, ^26^ ^]^ It catalyzes the cleavage of six‐carbon fructose 1,6‐bisphosphate (F1,6BP) into two three‐carbon metabolites, glyceraldehyde 3‐phosphate (G3P) and dihydroxyacetone phosphate (DHAP). This reaction is not only central to glycolysis but also connected to several vital metabolic pathways, including the pentose phosphate pathway (PPP), fatty acid and phospholipid synthesis, and serine biosynthesis. In addition, aldolase contributes to several significant signaling pathways, such as PI3K‐dependent glycolysis,^[^ ^27^ ^]^ and the glucose‐mediated modulation of mechanistic target of rapamycin complex 1 (mTORC1) and AMP‐activated protein kinase (AMPK) activities.^[^ ^28^ ^]^ Notably, among the aldolase family members (ALDOA, ALDOB, and ALDOC), ALDOA is the predominant isoform.^[^ ^24^ ^]^ It is highly expressed in many cancer types compared to corresponding normal tissues (Figure S1, Supporting Information), and exhibits the lowest Km for F1,6BP catalysis.^[^ ^24^ ^]^ In light of the above, ALDOA plays a significant role in reprogramming cancer metabolism.

Although targeting glycolytic enzymes preferentially exploited by cancer cells has been regarded as a promising therapeutic strategy, the adaptation and resistance of cancer cells to glycolysis inhibitors have severely hindered its application.^[^ ^23^ ^]^ Therefore, investigating whether any glycolytic enzyme could serve as targetable ferroptotic vulnerability in cancer cells, and then targeting such enzyme in conjugation with mild (i.e., non‐toxic to normal cells) ferroptosis induction could be a more effective and selective cancer therapy approach. Here, we identified ALDOA as a targetable vulnerability for ferroptosis sensitization in cancer. Genetic or pharmacological suppression of ALDOA can specifically promote ferroptosis in cancer cells, both in vitro and in mouse xenograft models. Notably, our findings highlight the molecular link between the Warburg effect and the ferroptosis‐suppressing mechanism, positioning ALDOA as a potential therapeutic target for ferroptosis‐mediated cancer treatment.

## 2 Results

### 2.1 ALDOA Represents a Targetable Vulnerability for Ferroptosis Sensitization in Cancer

**Figure 1:**
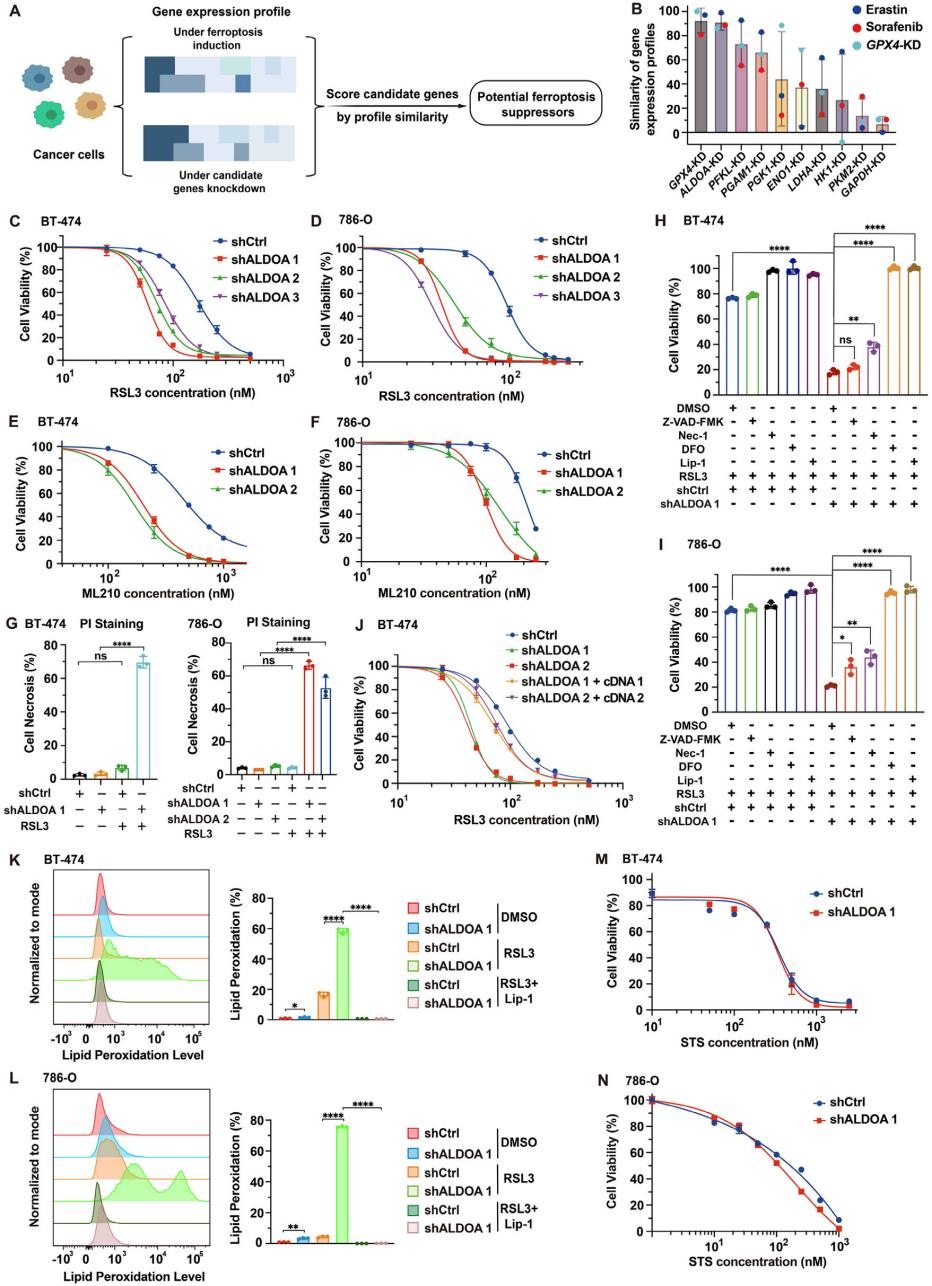
ALDOA depletion promotes ferroptosis susceptibility in cancer cells. A) Schematic of the virtual screening strategy. B) Connectivity Map (Touchstone) analysis showing the transcriptional similarity between knockdown (KD) signatures of core glycolytic enzymes and ferroptosis reference signatures (erastin, sorafenib, and GPX4 KD) across cancer cell lines. For each glycolytic‐gene KD signature, connectivity scores were computed against each ferroptosis reference signature; bars show the mean connectivity across inducers and error bars indicate the standard deviation (s.d.). Positive hits were defined as those with connectivity scores ≥ 80. C,D) Dose‐dependent toxicity of the ferroptosis inducers RSL3 in BT‐474 and 786‐O cells expressing untargeted shRNA (shCtrl) or shRNAs targeting *ALDOA* (shALDOA). Cell viability was assessed after 24 h of treatment. E,F) Dose‐dependent toxicity of the ferroptosis inducers ML210 in the indicated BT‐474 and 786‐O cells after 24 h of treatment. G) Quantification of necrotic cells by PI staining and flow cytometry after treatment with 100 nm RSL3 (BT‐474) or 50 nm RSL3 (786‐O) for 24 h. H,I) Viability of the indicated BT‐474 and 786‐O cells treated with H) 75 nm or I) 50 nm RSL3, respectively, for 24 h in the presence or absence of Lip‐1 (500 nm), DFO (100 µm), Z‐VAD‐FMK (20 µm), or Necrostatin‐1 (10 µm). J) Dose‐dependent toxicity of RSL3 in BT‐474 cells expressing shCtrl, shALDOA, or a combination of shALDOA and shALDOA‐resistant *ALDOA* cDNAs. Viability was measured after treatment with RSL3 for 24 h. K,L) Lipid peroxidation assessment of the indicated K) BT‐474 and L) 786‐O cells treated with K) 500 nm RSL3 or L) 200 nm RSL3, respectively, for 6 h, in the presence or absence of Lip‐1 (500 nm). M,N) Viability of the indicated BT‐474 and 786‐O cells with the treatment of indicated concentrations of staurosporine (STS) for 24 h. Data are mean ± s.d. of n = 3 biological replicates in (C–N). The data shown are representative of two (G,K,L) or three (C–F,H–J, M,N) independent experiments. Statistical analysis was performed using two‐way ANOVA (G) or two‐tailed unpaired Student's t‐test (H,I,K,L). ^*^ *p* < 0.05, ^**^ *p* < 0.01, ^****^ *p* < 0.0001. ns, not significant.

As ferroptosis sensitivity is heavily influenced by metabolic alterations,^[^ ^21^ ^]^ we explored the hypothesis that targeting enzymes involved in aerobic glycolysis‐a major metabolic reprogramming in cancer cells‐could enhance ferroptosis sensitivity specifically in cancer cells. To this end, we conducted a comparative analysis based on the Touchstone dataset from the Connectivity Map (CMap), which includes a collection of compound and genetic perturbagens that generate robust gene expression signatures in cells.^[^ ^29^ ^]^ Specifically, gene expression profiles from established ferroptosis‐inducing strategies (i.e., erastin or sorafenib treatment and *GPX4* knockdown) across all available cancer cell lines in the database were collected and compared with gene expression profiles from the knockdown of each individual glycolytic enzyme (**Figure** **1**A). Notably, among genes evaluated, ALDOA stood out as a potential target for ferroptosis sensitization in cancer (Figure 1B).

To further validate the role of ALDOA in ferroptosis regulation, we utilized specific shRNAs to knockdown *ALDOA* in breast carcinoma cell line BT‐474 and renal cell carcinoma cell line 786‐O (Figure S2A–D, Supporting Information). Cells expressing shRNAs targeting *ALDOA* (shALDOA) showed enhanced susceptibility to GPX4 inhibitors RSL3 and ML210, as well as system X_c_^−^ inhibitor imidazole ketone erastin (IKE) (Figure 1C–G; Figure S2E–G, Supporting Information). Notably, the enhanced sensitivity to RSL3 was rescued by canonical ferroptosis inhibitors liproxstatin‐1 (Lip‐1, a radical‐trapping antioxidant) or deferoxamine (DFO, an iron chelator), but not by the apoptosis inhibitor Z‐VAD‐FMK or the necroptosis inhibitor necrostatin‐1 (Nec‐1) (Figure 1H,I). Additionally, this increased susceptibility was reversed by reintroducing shRNA‐resistant cDNAs of *ALDOA* (Figure 1J; Figure S2H, Supporting Information). Moreover, BT‐474 and 786‐O cells with ALDOA depletion exhibited increased lipid peroxidation, a hallmark of ferroptosis, as monitored by BODIPY 581/591 C11 staining following RSL3 treatment (Figure 1K,L). Furthermore, these cells had no altered susceptibility to apoptosis inducer staurosporine (Figure 1M,N). Collectively, these data demonstrate that ALDOA depletion specifically enhances the susceptibility of BT‐474 and 786‐O cancer cells to ferroptosis.

In addition to BT‐474 and 786‐O cells, we further confirmed the role of ALDOA depletion in ferroptosis sensitization in a wider range of cancer cells, including another invasive breast carcinoma cell line T‐47D, a pleural mesothelioma cell line NCIH‐226, and a neuroblastoma cell line SK‐N‐DZ (Figure S3A–K, Supporting Information). Moreover, upon mild ferroptosis induction, ALDOA‐depleted 786‐O and T‐47D cells, but not control cells, displayed characteristic morphological features of ferroptosis, including mitochondrial shrinkage and cristae loss (Figure S3L,M, Supporting Information). These results indicate that ALDOA represents a targetable ferroptosis vulnerability in a broad type of cancer cells.

In remarkable contrast, ALDOA depletion did not promote ferroptosis susceptibility in noncancerous (normal) cells such as human mammary epithelial cell line MCF10A, human fibroblast cell lines HFF‐1 (human foreskin fibroblast) and HFL‐1 (human lung fibroblast), and human retinal pigmented epithelial cell line ARPE‐19 (Figure S4A–D, Supporting Information). Additionally, ALDOA depletion has no promoting effect on lipid peroxidation level in MCF10A and HFF‐1 cells with RSL3 treatment (Figure S4E, Supporting Information). Altogether, these results validated that ALDOA is a cancer‐specific vulnerability for ferroptosis sensitization in vitro.

**Figure 2:**
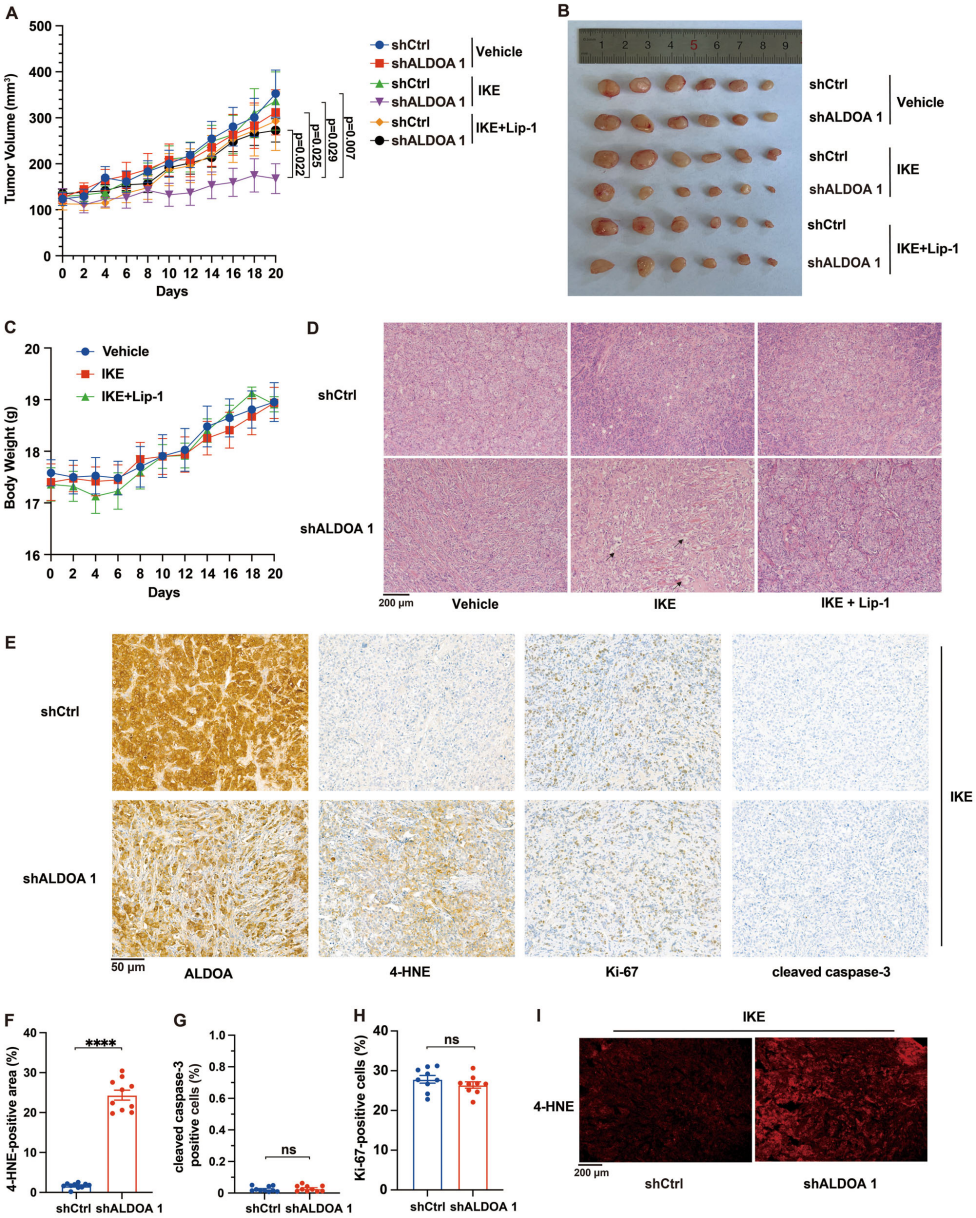
ALDOA depletion promotes ferroptosis in cancer xenografts. A) Tumor growth rates of BT‐474 xenografts expressing shCtrl or shALDOA with the indicated treatments over time. IKE (40 mg kg^−1^), Lip‐1 (10 mg kg^−1^), intraperitoneally, once every day. Data are mean ± s.e.m. from two independent experiments. n=10 in IKE and Lip‐1 co‐treatment (IKE + Lip‐1) group and n=11 in the other two groups. To compare between groups of mice at the experimental endpoint, statistical analysis was performed using the two‐tailed unpaired Student's t‐test. shALDOA+IKE compared with shALDOA+Vehicle, P=0.025; shALDOA+IKE compared with shCtrl+IKE, P=0.029; shALDOA+IKE compared with shCtrl+Vehicle, P = 0.007; shALDOA+IKE compared with shALDOA+IKE+Lip‐1, P = 0.022. B) Images of resected tumors from mice xenografted with control and ALDOA‐depleted BT‐474 cells in one of the two independent experiments. C) Body weight of mice xenografted with control and ALDOA‐depleted BT‐474 cells, respectively, into the left and right flanks under the indicated treatments over time. n = 10 in IKE+Lip‐1 group and n = 11 in the other two groups. Data plotted are mean ± s.e.m. D) Representative H&E staining in paraffin section of the indicated BT‐474 xenografts with indicated treatments. Scale bar, 200 µm. E) Representative immunohistochemical staining of 4‐HNE, Ki‐67, and cleaved caspase‐3 in paraffin section of the indicated BT‐474 xenografts following IKE treatment. Scale bar, 50 µm. F–H) Quantification of immunohistochemical staining of F) 4‐HNE, G) cleaved caspase‐3, and H) Ki‐67 staining in BT‐474 xenografts with the indicated treatments. Data are mean ± s.d. (n = 10). Statistical analysis was performed using two‐tailed unpaired Student's t‐test. ****P < 0.0001. ns, not significant. I) Representative immunohistochemical fluorescence imaging of 4‐HNE in the indicated BT‐474 xenografts following IKE treatment. Scale bar, 200 µm.

We subsequently assessed the therapeutic potential of targeting ALDOA using a preclinical xenograft tumor model. To this end, we subcutaneously injected control and ALDOA‐depleted BT‐474 cells, respectively, into the left and right flanks of athymic nude mice, and examined the effect of IKE, an erastin derivative amenable for use in vivo, on tumor growth. Our data showed that the volume of tumors grown from ALDOA‐depleted BT‐474 cells was reduced by treatment with a mild dose of IKE, which was reversed by coadministration of Lip‐1. In contrast, the volume of tumors grown from control BT‐474 cells was unaffected (**Figure** **2**A,B). Importantly, there was no significant body weight loss during the entire drug administration period (Figure 2C), indicating the appropriate dosing regimen. Further analyses revealed that necrosis areas and lipid peroxidation marker 4‐hydroxynonenal (4‐HNE) were both enriched in ALDOA‐depleted tumors following IKE treatment (Figure 2D–F,I). In contrast, the apoptosis marker cleaved caspase‐3 and the proliferation marker Ki‐67 remained largely unchanged (Figure 2E,G,H). Collectively, these results demonstrate that ALDOA suppression promotes ferroptosis in cancer cells both in vitro and in vivo, establishing ALDOA as a targetable vulnerability for ferroptosis sensitization in cancer.

### 2.2 ALDOA Controls Ferroptosis Vulnerability in Cancer Cells via Metabolic Reprogramming

**Figure 3:**
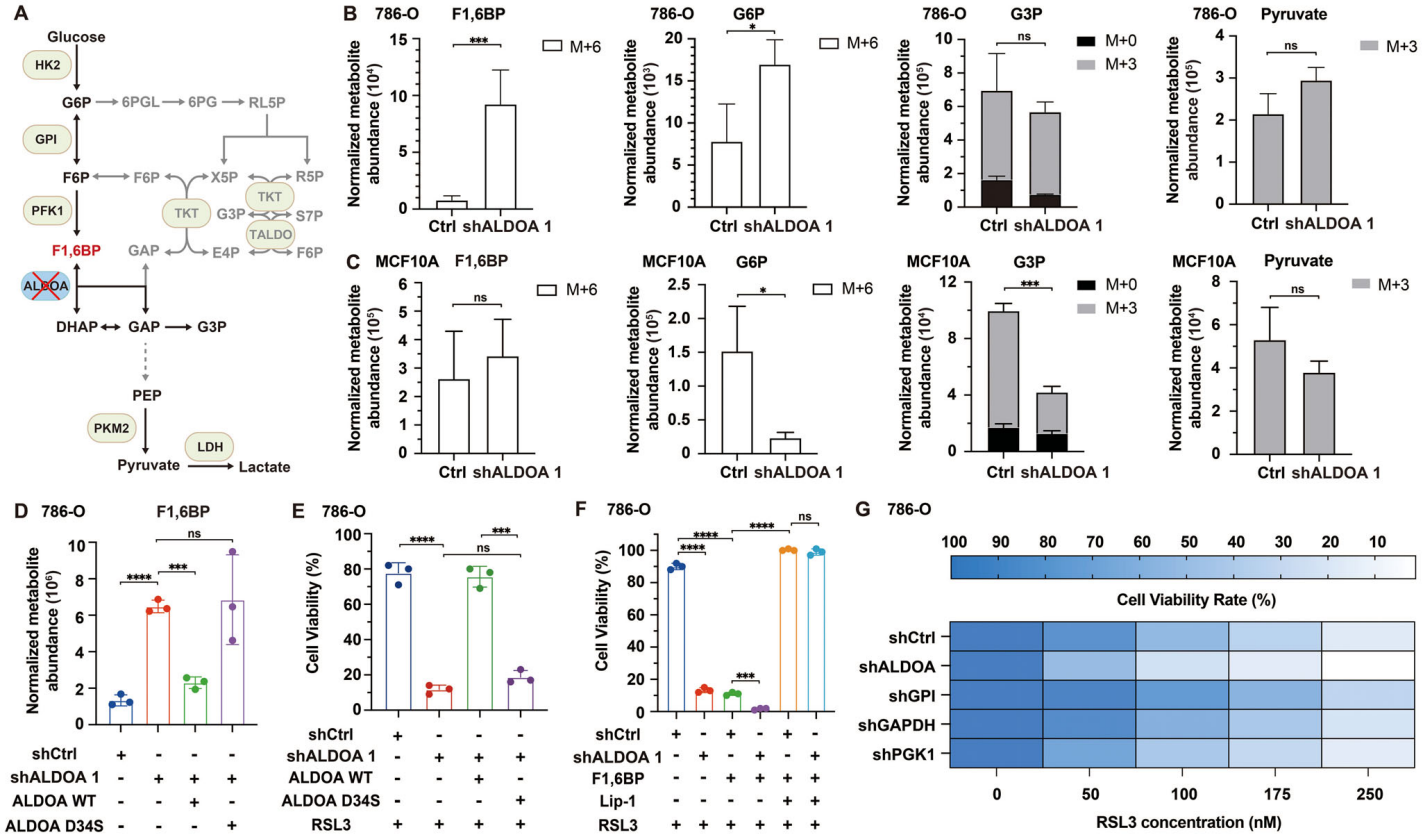
ALDOA regulates ferroptosis through metabolic reprogramming. A) Schematic of glycolysis and pentose phosphate shunt indicating disruption of ALDOA. 6PG, 6‐phosphogluconate; 6PGL, 6‐phosphogluconolactone; ALDOA, aldolase A; DHAP, dihydroxyacetone phosphate; E4P, erythrose 4‐phosphate; F1,6BP, fructose 1,6­‐bisphosphate; F6P, fructose 6­‐phosphate; G3P, glycerol 3‐­phosphate; G6P, glucose 6‐­phosphate; GAP, glyceraldehyde 3‐­phosphate; GPI, glucose phosphate isomerase; HK2, hexokinase 2; LDH, lactate dehydrogenase; PEP, phosphoenolpyruvate; PFK1, phosphofructokinase‐­1; PKM2, pyruvate kinase M2; R5P, ribose 5‐phosphate; RL5P, ribulose 5‐phosphate; S7P, sedoheptulose 7‐phosphate; TALDO, transaldolase; TKT, transketolase; X5P, xylulose 5‐phosphate. B,C) Labeled and unlabeled metabolite levels for F1,6BP, G6P, G3P, and pyruvate in (B) 786O or C) MCF10A cells expressing either shCtrl or shALDOA. Cells were cultured in full medium or glucose‐deficient medium containing U‐^13^C‐glucose for 0.5 h. Natural isotope corrected isotopologue abundances normalized to biomass are shown. D) Targeted metabolomic quantification of F1,6BP in 786‐O cells expressing shCtrl, shALDOA, or shALDOA complemented with WT or D34S ALDOA. E) Cell viability of 786‐O cells expressing shCtrl, shALDOA, or shALDOA complemented with WT or D34S ALDOA following treatment with 50 nm RSL3 for 24 h. F) Viability of the ALDOA‐depleted and control 786‐O cells treated with 50 nm RSL3 for 24 h, with or without co‐treatment with 1 mm F1,6BP. Lip‐1 (500 nm) was used to rescue ferroptosis. G) Heatmap showing the viability of 786‐O cells expressing shCtrl, shALDOA, shGPI, shGAPDH, or shPGK1 treated with the indicated concentration of RSL3 for 24 h. Data are mean ± s.d. of n = 3 biological replicates in (B–G). Data are representative of E–G) two independent experiments and B–D) one experiment. Cell viability following RSL3 treatment was normalized to the corresponding DMSO‐treated controls in (E,F). Statistical analysis was performed using two‐tailed unpaired Student's t‐test in (D–F). ^*^ *p* < 0.05, ^***^ *p* < 0.001, ^****^ *p* < 0.0001. ns, not significant.

Given ALDOA's key role in glycolysis and its broad impact on cellular metabolism,^[^ ^24^ ^]^ we subsequently conducted a [U‐­^13^C­]glucose tracing assay coupled with liquid chromatography‐mass spectrometry (LC‐MS) analysis to measure glucose metabolism in ALDOA‐depleted and control 786‐O cells (**Figure** **3**A). Notably, upstream glycolytic metabolites F1,6BP and G6P accumulated in ALDOA‐depleted cells, whereas metabolites of the lower glycolysis were only mildly affected (Figure 3B; Figure S5A, Supporting Information). In contrast, ALDOA depletion in noncancerous human mammary epithelial MCF10A cells did not result in F1,6BP accumulation and instead led to a reduction in G6P levels, suggesting a decreased rate of upstream glycolysis in these cells (Figure 3C; Figure S5B, Supporting Information). This divergent response indicates that cancer cells rewire glycolysis to sustain flux under ALDOA depletion, resulting in the buildup of upstream intermediates.

To determine whether this glycolytic reprogramming regulates ferroptosis in cancer cells, we first examined whether ALDOA's enzymatic activity is required. Reintroduction of wild‐type ALDOA into ALDOA‐depleted 786‐O cells reversed the accumulation of F1,6BP and rescued the enhanced ferroptosis sensitivity, whereas reintroduction of a catalytically inactive ALDOA mutant (D34S) failed to do so (Figure 3D,E; Figure S5C, Supporting Information). These results indicate that ALDOA's enzymatic function is essential for ferroptosis regulation. To determine whether F1,6BP accumulation mediates ferroptosis sensitization in ALDOA‐depleted cells, we supplemented 786‐O cells with exogenous F1,6BP and observed a significant increase in ferroptosis sensitivity (Figure 3F). Accordingly, ameliorating the accumulation of F1,6BP by suppressing the upstream enzyme GPI (glucose phosphate isomerase) reverses the enhanced ferroptosis sensitivity in ALDOA‐depleted 786‐O cells (Figure S5D, Supporting Information). Moreover, silencing glycolytic enzymes adjacent to ALDOA failed to elicit ferroptosis sensitivity comparable to that induced by ALDOA depletion in 786‐O cells (Figure 3G). Collectively, these findings demonstrate that ALDOA depletion reprograms metabolism and drives F1,6BP accumulation in cancer cells, thereby sensitizing these cells to ferroptosis.

### 2.3 ALDOA Regulates Ferroptosis Sensitivity through Phospholipid Remodeling

**Figure 4:**
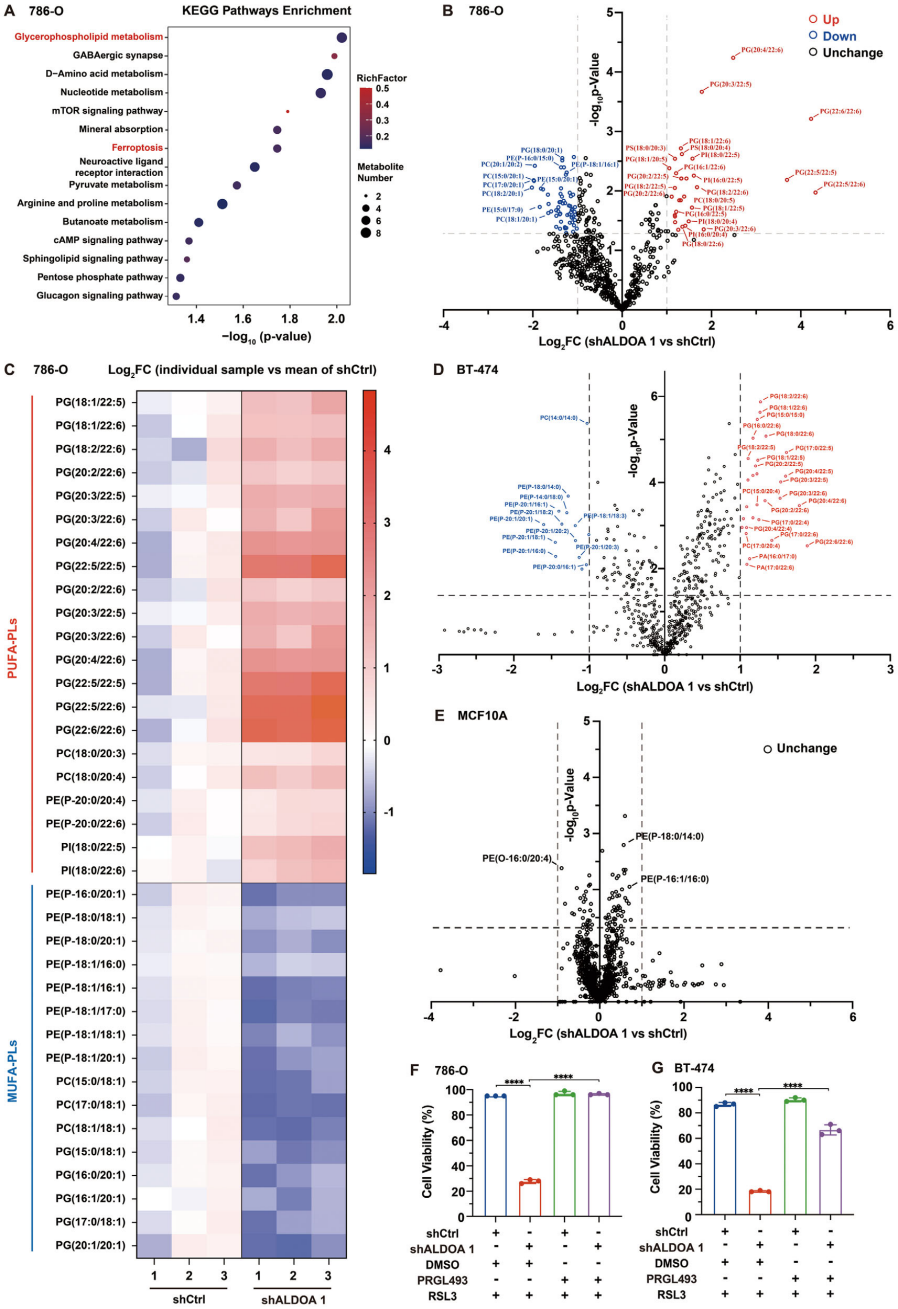
ALDOA regulates ferroptosis through phospholipid remodeling. A) KEGG enrichment analysis of the differentially abundant metabolites between control and ALDOA‐depleted 786‐O cells. The glycerophospholipid metabolism and ferroptosis pathways are highlighted in red. B) Volcano plot showing upregulated (red) and downregulated (blue) lipid species following ALDOA depletion in 786‐O cells. Cutoff: FC threshold = 2, p < 0.05; two‐tailed t test. C) Heatmap showing the normalized abundances of significantly altered PUFA‐phospholipids and MUFA‐phospholipids between control and ALDOA‐depleted 786‐O cells. D) Volcano plot displaying lipidomic changes upon ALDOA depletion in BT‐474 cells. Cutoff: FC threshold = 2, p < 0.05; two‐tailed t test. E) Volcano plot showing the lipidomic changes upon ALDOA depletion in noncancerous MCF10A cells. Cutoff: FC threshold = 2, p < 0.05; two‐tailed t test. F,G) Cell viability of the indicated F) 786‐O and G) BT‐474 cells treated with F) 50 nm RSL3 or G) 75 nm RSL3 for 24 h, with or without 12 h pre‐treatment using the ACSL4 inhibitor PRGL493 (5 µm). Cell viability following RSL3 treatment was normalized to the corresponding DMSO‐treated controls: the viability of DMSO‐treated shCtrl cells was defined as 100% to calculate the relative viability of RSL3‐treated shCtrl cells, and the viability of DMSO‐treated shALDOA cells was similarly set as 100% for normalization of RSL3‐treated shALDOA cells. Data are from n = 3 biological replicates in (A–G) and are presented as mean ± s.d. in (F,G). Data are representative of (F,G) three independent experiments or (A–E) one experiment. Statistical analysis was performed using two‐way ANOVA in (F,G). ^****^ *p* < 0.0001.

In order to reveal the mechanisms underlying ALDOA‐mediated ferroptosis protection in cancer cells, we conducted untargeted metabolomics analyses on ALDOA‐depleted and control 786‐O cancer cells. Notably, KEGG analysis revealed significant enrichment of glycerophospholipid metabolism and ferroptosis pathways among differential metabolites between ALDOA‐depleted and control cells (**Figure** **4**A). To further explore the phospholipid alterations in ALDOA‐depleted cancer cells, we conducted a quantitative lipidomic analysis on ALDOA‐depleted and control 786‐O cells. As expected, while the overall content of phospholipids remained largely unchanged (Figure S6A, Supporting Information), ALDOA depletion led to a marked increase in a broad spectrum of PUFA‐containing phospholipids (PUFA‐PLs), including PUFA‐containing phosphatidylglycerols (PGs), phosphatidylcholines (PCs), phosphatidylethanolamines (PEs), and phosphatidylinositols (PIs) (Figure 4B,C; Figure S6B–E, Supporting Information). Among these, PUFA‐PGs, especially PG (22:6,22:6), were the most enriched (Figure 4B,C). These phospholipids with two PUFA chains have recently been identified as highly vulnerable to ferroptosis induction.^[^ ^30^ ^]^ Besides, along with the accumulation of PUFA‐PLs, ALDOA depletion resulted in a significant reduction in anti‐ferroptosis monounsaturated fatty acid‐containing phospholipids (MUFA‐PLs), including MUFA‐PEs, MUFA‐PGs, and MUFA‐PCs (Figure 4B,C; Figure S6B–E, Supporting Information). Similarly, the enrichment of PUFA‐PLs and depletion of MUFA‐PLs were also observed in ALDOA‐depleted BT‐474 cancer cells (Figure 4D). In contrast, ALDOA depletion has a negligible effect on phospholipids remodeling in the human mammary epithelial cell line MCF10A (Figure 4E), indicating the distinct phospholipid reprogramming between cancerous and noncancerous cells in response to ALDOA depletion. Furthermore, the increased sensitivity to RSL3 in ALDOA‐depleted 786‐O and BT‐474 cells was markedly suppressed by treatment with the ACSL4 inhibitor RPGL493 (Figure 4F,G). RPGL493 blocks the conversion of PUFAs into their active acyl‐CoA derivatives, thereby impeding the incorporation of PUFAs into phospholipids for PUFA‐PLs synthesis. This finding further supports that ferroptosis sensitization upon ALDOA depletion is driven by the shift in the PUFA‐PLs/MUFA‐PLs ratio resulting from phospholipid remodeling. Collectively, these results demonstrate that ALDOA suppression reprograms lipid metabolism toward a pro‐ferroptotic phospholipid profile, rendering cancer cells more susceptible to ferroptosis.

### 2.4 ALDOA Depletion–Induced Metabolic Reprogramming Modulates Phospholipid Composition through Autophagy Activation and Ubiquitination‐Dependent Degradation of Phospholipid‐Modifying Enzymes

**Figure 5:**
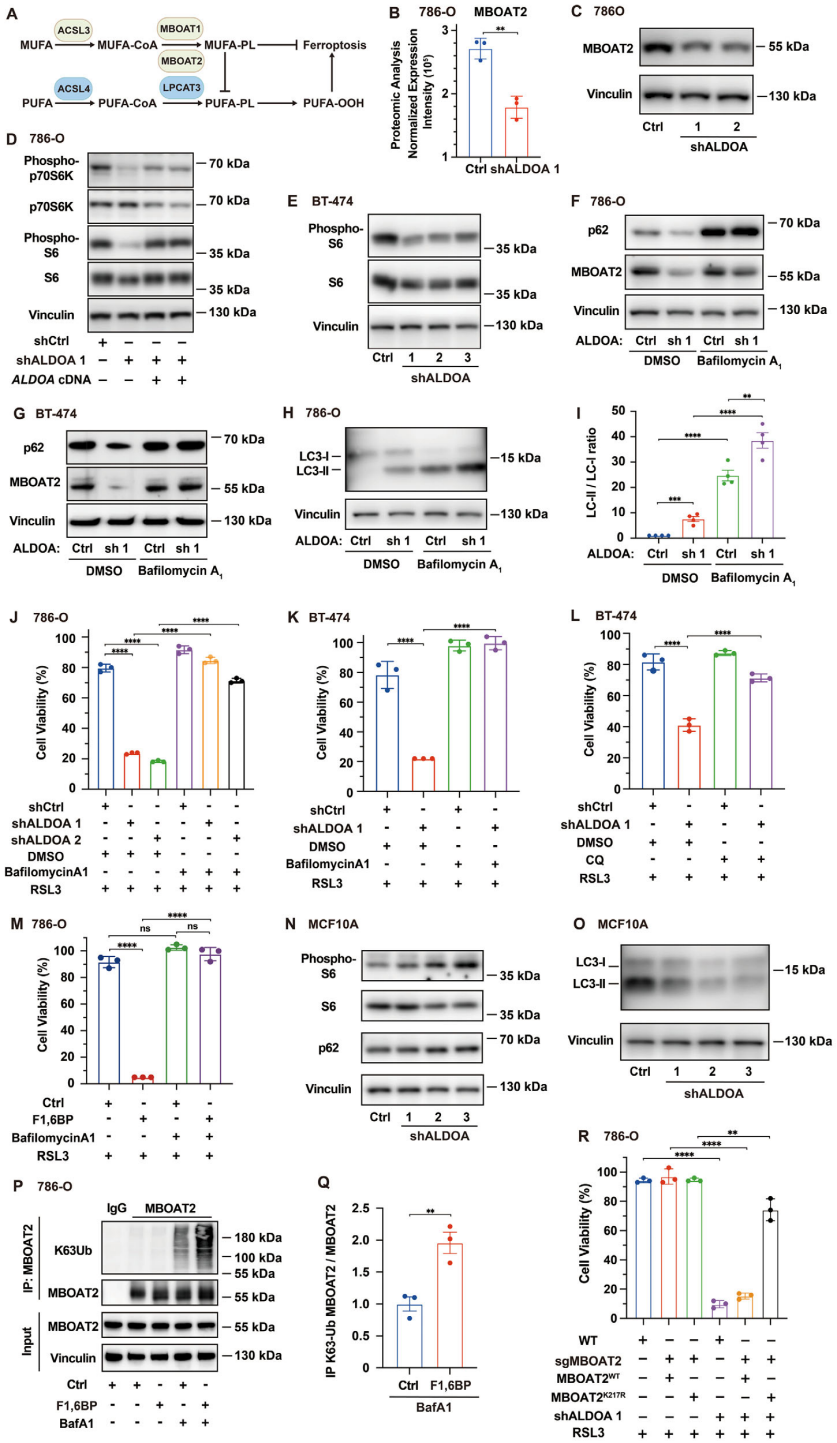
ALDOA depletion induces phospholipid remodeling through ubiquitination‐dependent autophagic degradation of MBOAT2. A) Schematic of the key enzymes involved in phospholipid remodeling. ACSL3/4, acyl‐CoA synthetase long‐chain family member 3/4; MBOAT1/2, membrane‐bound O‐acyltransferase domain‐containing 1/2; LPCAT3, lysophosphatidylcholine acyltransferase 3. B,C) Protein levels of MBOAT2 in control and ALDOA‐depleted 786‐O cells measured by B) proteomic and C) Western blot analysis. D,E) mTORC1 activity indicated by the phosphorylation of ribosome S6 (S6) and p70 ribosomal protein S6 kinase (p70S6K) in the indicated D) 786‐O and E) BT‐474 cells. F,G) Autophagic flux and MBOAT2 degradation in ALDOA‐depleted and control F) 786‐O and G) BT‐474 cells, with or without pretreatment with 100 nm Bafilomycin A1 for 12 h. H,I) Western blot (H) and quantification (I) analyses of autophagic flux in control and ALDOA‐depleted 786‐O cells, as indicated by LC3 turnover, with or without 100 nm Bafilomycin A1 pretreatment for 12 h. J,K) Viability of the indicated 786‐O and BT‐474 cells under the treatment with J) 50 nm and K) 75 nm RSL3 for 24 h, with or without pretreatment with J) 50 nm or K) 100 nm Bafilomycin A1 for 12 h. L) Viability of the indicated BT‐474 cells treated with 75 nm RSL3 for 24 h, with or without pretreatment with 10 µm chloroquine (CQ) for 12 h. M) Viability of the control and F1,6BP (1 mm)‐treated 786‐O cells exposed to 50 nm RSL3 for 24 h, with or without pretreatment with 50 nm Bafilomycin A1 for 12 h. N,O) mTORC1 activity indicated by the phosphorylation of ribosome S6 (N), and autophagy activity indicated by N) p62 degradation and O) LC3 turnover in MCF10A cells under control and ALDOA‐depleted conditions. P,Q) Western blot (P) and quantification (Q) analyses of K63‐linked polyubiquitination on immunoprecipitated (IP) MBOAT2 in 786‐O cells stably overexpressing MBOAT2, with or without 5 mm F1,6BP pretreatment for 6 h. Bafilomycin A1 (100 nm, 4 h before harvest) was used to inhibit autophagy‐dependent degradation. IgG served as a negative control for MBOAT2 antibody. R) Viability of the WT and MBOAT2‐KO (sgMBOAT2) 786‐O cells re‐expressing WT or K217R mutant MBOAT2, with or without ALDOA‐depletion (shALDOA 1), following treatment with 50 nm RSL3 for 24 h. Data are mean ± s.d. of n = 3 biological replicates in (B,I–M,Q,R). Statistical analysis was performed using two‐tailed unpaired Student's t‐test in (B,I,Q,R) or two‐way ANOVA in (J–M). ^**^ *p* < 0.01, ^***^ *p* < 0.001, ^****^ *p* < 0.0001. ns, not significant.

To decipher the mechanism underlying phospholipid remodeling in cancer cells with ALDOA suppression, we first examined enzymes involved in phospholipid modification (**Figure** **5**A). Proteomic and Western blot analyses revealed that ALDOA depletion markedly reduced membrane‐bound O‐acyltransferase domain‐containing 1 and 2 (MBOAT1/2) expression in 786‐O cells, whereas ACSL4 and LPCAT3 remained largely unchanged (Figure 5B,C; Figure S7A–D, Supporting Information). Consistently, MBOAT1 and MBOAT2 were also downregulated in BT‐474 cells upon ALDOA depletion (Figure S7E,F, Supporting Information). MBOAT1 and MBOAT2 have been reported to regulate phospholipid composition and ferroptosis sensitivity, with MBOAT2 overexpression conferring greater resistance to ferroptosis than MBOAT1.^[^ ^31^ ^]^ Across cell lines used in this study, the basal expression of MBOAT1 was much lower than that of MBOAT2 (Figure 5B; Figure S7A,G, Supporting Information), suggesting that downregulation of MBOAT2, rather than MBOAT1, primarily accounts for the global phospholipid remodeling phenotype observed in ALDOA‐depleted cancer cells. Consistently, ALDOA depletion failed to further enhance ferroptosis in MBOAT2‐knockout 786‐O cells, while re‐expression of MBOAT2 in ALDOA‐depleted 786‐O cells largely reversed the enhanced ferroptosis sensitivity induced by ALDOA depletion (Figure S7H–J, Supporting Information). Together, these data identify MBOAT2 downregulation as the key downstream effector through which ALDOA suppression reprograms the phospholipid profile and enhances ferroptosis sensitivity in cancer cells.

Given that the reduction of MBOAT2 occurs at only the post‐transcriptional level (Figure S7K, Supporting Information), and ALDOA inhibition has previously been reported to suppress mTORC1 activity and induce autophagy,^[^ ^28^, ^32^ ^]^ we next examined whether autophagy contributes to MBOAT2 downregulation. Indeed, ALDOA depletion reduced mTORC1 signaling in 786‐O and BT‐474 cells, which was reversed upon re‐expressing *ALDOA* cDNA in 786‐O cells (Figure 5D,E). In addition, these ALDOA‐depleted cancer cells consistently exhibit enhanced autophagic activities in terms of decreased p62 (SQSTM1) levels, increased conversion of cytosolic microtubule‐associated protein 1 light chain 3 (LC3‐I) into membrane‐associated LC3‐II, and elevated LysoTracker puncta (Figure 5F–I; Figure S8A, Supporting Information). Furthermore, alterations in these canonical autophagy markers^[^ ^33^ ^]^ were effectively reversed by the lysosomal inhibitor bafilomycin A1 (Figure 5F–I; Figure S8A, Supporting Information). Meanwhile, the decreased abundance of MBOAT2 in ALDOA‐depleted 786‐O and BT‐474 cells was also restored by treatment with bafilomycin A1 (Figure 5F,G), collectively suggesting that the reduction of this MUFA‐PL–synthesizing enzyme is mediated by autophagy‐dependent degradation. Functionally, the ferroptosis sensitivity induced by ALDOA depletion in BT‐474 and 786‐O cells was reversed by pharmacological inhibition of the autophagy–lysosome pathway using bafilomycin A1 or chloroquine (Figure 5J–L). Likewise, the enhanced ferroptosis sensitivity induced by F1,6BP in 786‐O cells was also reversed by bafilomycin A1 (Figure 5M). To assess whether this effect is specific to MBOAT2, we next examined other canonical ferroptosis regulators. The level of nuclear receptor coactivator 4 (NCOA4) was not significantly altered in ALDOA‐depleted 786‐O cells, indicating that ferritinophagy is not a major contributor to ferroptosis sensitization in this context (Figure S9A,B, Supporting Information). Consistently, the abundance of established ferroptosis suppressors, including GPX4, SLC7A11, FSP1, and DHODH, remained unchanged in 786‐O and BT‐474 cells following ALDOA depletion (Figure S9C–G, Supporting Information), further excluding those as mediators of the observed phenotype. Collectively, these results demonstrate that ALDOA depletion induces autophagy activation and the subsequent degradation of MBOAT2 in cancer cells.

Given that metabolic reprogramming is required for ALDOA depletion to regulate ferroptosis sensitivity, we next investigated whether perturbation of ALDOA enzymatic activity drive this autophagy‐dependent phospholipid remodeling. To test this, we introduced wild‐type ALDOA or the catalytically inactive D34S mutant into ALDOA‐depleted 786‐O cells and found that wild‐type, but not D34S, restored MBOAT2 levels, indicating that ALDOA enzymatic activity is required to maintain MBOAT2 stability (Figure S10A, Supporting Information). Consistently, exogenous F1,6BP suppressed mTORC1 signaling and activated autophagy in 786‐O cells, accompanied by a reduction in MBOAT2, phenocopying ALDOA depletion (Figure S10B,C, Supporting Information). These data collectively show that F1,6BP accumulation upon ALDOA loss is sufficient to activate autophagy and the subsequent degradation of MBOAT2. In contrast, in the noncancerous mammary epithelial cell line MCF10A, where ALDOA depletion did not cause detectable accumulation of F1,6BP, mTORC1 activity remained unchanged after ALDOA suppression (Figure 5N). Meanwhile, p62 (SQSTM1) levels remained stable and the conversion of LC3‐I to LC3‐II was inhibited, both indicating that autophagy was not induced in these noncancerous cells (Figure 5N,O). This differential response between cancerous and noncancerous cells further confirms that ALDOA depletion–mediated metabolic rewiring acts as the principal regulator of MBOAT2‐dependent phospholipid remodeling selectively in cancer cells.

To further investigate how MBOAT2 is targeted for degradation following ALDOA depletion in cancer cells, we performed mass spectrometry–based ubiquitination profiling on immunoprecipitated MBOAT2. This analysis identified lysine 217 (K217) of MBOAT2 as a ubiquitination site and revealed that MBOAT2 undergoes K63‐linked ubiquitination (Figure S10D,E, Supporting Information). Notably, F1,6BP treatment markedly enhanced the K63‐linked ubiquitination of MBOAT2 in 786‐O cancer cells (Figure 5P,Q). We next investigated whether K63‐linked polyubiquitination at K217 dictates the autophagy‐dependent degradation of MBOAT2. Reintroduction of MBOAT2 K217R mutant cDNAs in MBOAT2‐KO cells, but not wild‐type MBOAT2, conferred resistance to ALDOA‐depletion‐induced MBOAT2 degradation and ferroptosis sensitization (Figure 5R; Figure S10F,G, Supporting Information). These results collectively indicate that ALDOA‐depletion‐mediated F1,6BP accumulation promotes K63‐linked ubiquitination and autophagy‐dependent degradation of MBOAT2, thereby sensitizing cancer cells to ferroptosis.

Collectively, ALDOA suppression–induced metabolic reprogramming activates autophagy and promotes the ubiquitination‐dependent degradation of MBOAT2, thereby shifting the lipid composition toward a pro‐ferroptotic state and sensitizing cancer cells to ferroptosis.

### 2.5 Pharmacological Inhibition of ALDOA Selectively Promotes Ferroptosis and Exhibits Therapeutic Potential in Cancer

**Figure 6:**
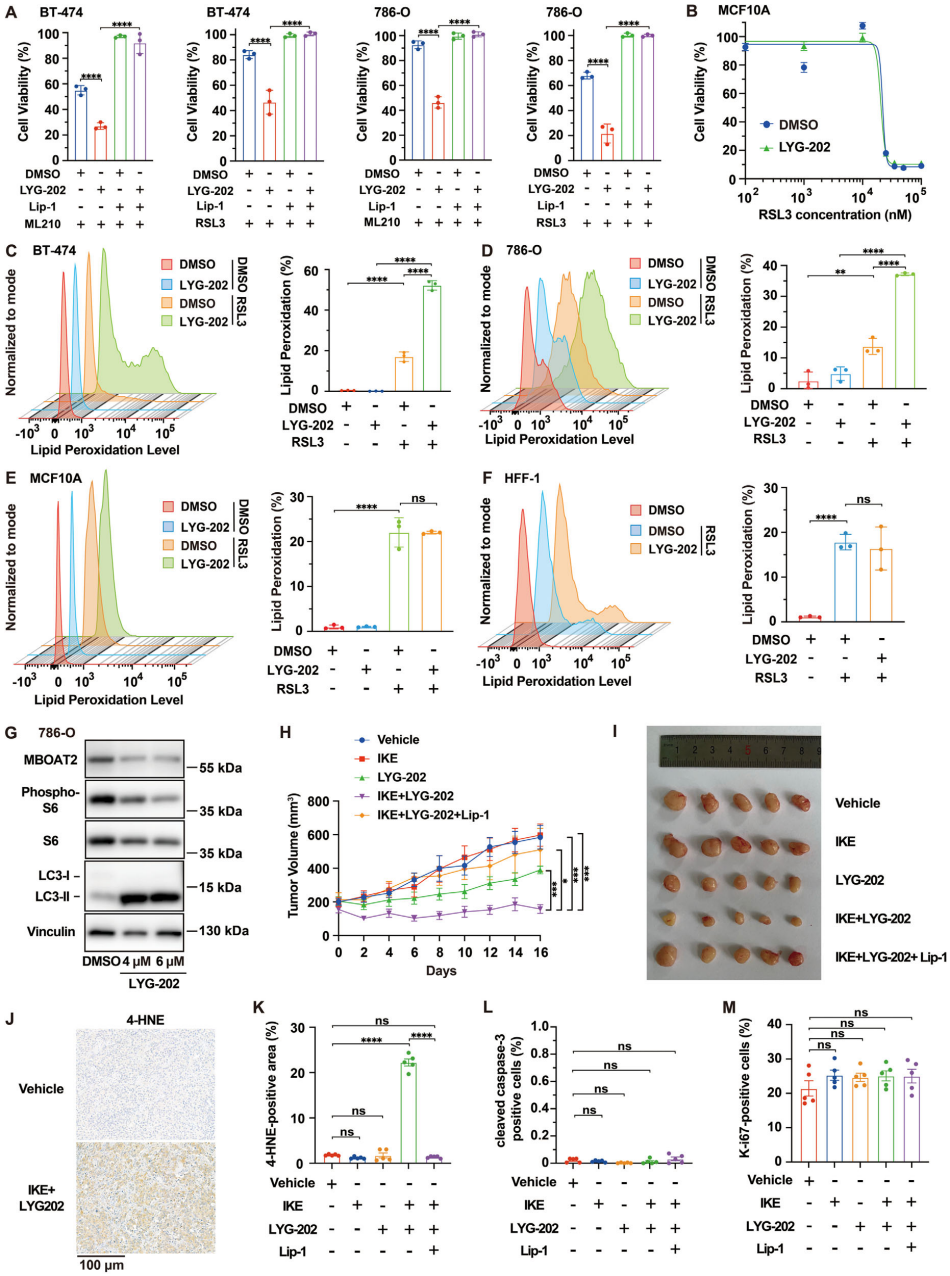
Pharmacological inhibition of ALDOA selectively promotes ferroptosis and suppresses tumor growth in cancer models. A) Viability of BT‐474 and 786‐O cells treated with ML210 (500 nm for BT‐474 and 100 nm for 786‐O) or RSL3 (100 nm for BT‐474 and 75 nm for 786‐O) for 24 h, with or without 24 h pretreatment with the ALDOA inhibitor LYG‐202 (4 µm). B) Viability of MCF10A cells treated with the indicated concentrations of RSL3 for 24 h, with or without 24 h pretreatment with 4 µm LYG‐202. C) Lipid peroxidation assessment of BT‐474 cells that were treated with 500 nm RSL3 for 6 h, with or without pretreatment with 4 µm LYG‐202 for 24 h. D) Lipid peroxidation assessment of 786‐O cells that were treated with 200 nm RSL3 for 4 h, with or without pretreatment with 4 µm LYG‐202 for 24 h. E) Lipid peroxidation assessment of MCF10A cells that were treated with 20 µm RSL3 for 12 h, with or without pretreatment with 4 µm LYG‐202 for 24 h. F) Lipid peroxidation assessment of HFF‐1 cells that were treated with 50 nm RSL3 for 8 h, with or without pretreatment with 4 µm LYG‐202 for 24 h. G) Detection of autophagy and mTORC1 activity as separately indicated by LC3 turnover and S6 phosphorylation, as well as the content of MBOAT2 in LYG‐202 treated 786‐O cells. H) Tumor growth curves of BT‐474 xenografts with the indicated treatments over time, IKE (80 mg kg^−1^), LYG‐202 (50 mg kg^−1^), Lip‐1 (10 mg kg^−1^), intraperitoneally, once every other day. n=6 in IKE+LYG‐202 group, and n = 5 in the other groups. Data are mean ± s.e.m. To compare between groups of mice at the experimental endpoint, P values were calculated using two‐tailed unpaired Student's t‐test. I) Images of resected tumors from BT‐474 cell‐xenografted mice with the treatment of the indicated small molecules. J) Representative immunohistochemical staining of 4‐HNE in paraffin section of the BT‐474 xenografts co‐treated with IKE and LYG‐202. Scale bar, 100 µm. K–M) Quantification of immunohistochemical staining for K) 4‐HNE, L) cleaved caspase‐3, and M) Ki‐67 in BT‐474 xenografts with the indicated treatments. Data are mean ± s.d. of n = 3 biological replicates in (A–F) and n = 5 in (K–M). Statistical analysis was performed using two‐way ANOVA in (A,C–E), one‐way ANOVA in (K–M), and two‐tailed unpaired Student's t‐test in (F). Data are representative of three independent experiments (A‐D), two independent experiments (E–G), and one experiment (H–M). ^*^ *p* < 0.05, ^**^ *p* < 0.01, ^***^ *p* < 0.001, ^****^ *p* < 0.0001. ns, not significant.

To analyze whether targeting ALDOA could provide therapeutic benefits to cancer patients, we examined clinical data from the GEPIA2 database. This analysis clearly shows that *ALDOA* expression is higher in various cancers compared to corresponding normal tissues (Figure S1A, Supporting Information) and that there is positive correlation between ALDOA expression and poor prognosis in patients across different cancer types (Figure S11A, Supporting Information). Given our findings on ALDOA's role in suppressing ferroptosis, we sought to examine whether small molecule inhibitors targeting ALDOA could offer significant therapeutic potential. Toward this end, the recently developed ALDOA inhibitor, LYG‐202,^[^ ^32^ ^]^ was first used to assess its effects on ferroptosis in vitro. We found that LYG‐202 increased the susceptibility of BT‐474 and 786‐O cells to ML210 or RSL3 induced ferroptosis (**Figure** **6**A). In contrast, LYG‐202 had minimal to no effect on STS‐induced apoptosis (Figure S11B, Supporting Information), confirming its specificity for ferroptosis regulation. Additionally, we demonstrated that aldometanib, another recently developed ALDOA inhibitor,^[^ ^34^ ^]^ also enhances ferroptosis susceptibility in 786‐O and BT‐474 cells (Figure S11C,D, Supporting Information). Importantly, ALDOA inhibitor enhanced ferroptosis sensitivity specifically in cancer cells, but not in various noncancerous cells, including MCF10A, ARPE‐19, HFL‐1, and HFF‐1 (Figure 6B; Figure S11E–G, Supporting Information). Furthermore, enhanced lipid peroxidation upon LYG‐202 and RSL3 co‐treatment was observed in cancer cells (BT‐474, 786‐O, T‐47D), but not in noncancerous cells (MCF10A, ARPE‐19) (Figure 6C–F; Figure S11H,I, Supporting Information). Consistent with ALDOA depletion, LYG‐202 treatment inhibited mTORC1 activity and enhanced LC3‐I to LC3‐II conversion in 786‐O cells (Figure 6G). This was also accompanied by a reduction in MBOAT2 levels (Figure 6G), mirroring the phenotype observed in ALDOA‐depleted cells (Figure 5C–I). Likewise, treatment with aldometanib also reduced mTORC1 activity in both 786‐O and BT‐474 cells (Figure S11J,K, Supporting Information). Collectively, ALDOA inhibitors exhibit effects similar to *ALDOA* knockdown, enhancing ferroptosis sensitivity specifically in cancer cells.

Next, we examined the therapeutic potential of LYG‐202 in mouse xenograft models. Compared to vehicle, LYG‐202 or IKE treated controls, the combination of LYG‐202 and IKE significantly reduced the growth of xenograft tumors derived from BT‐474 cells. This alteration could be largely reversed by co‐treatment with Lip‐1 (Figure 6H,I). There was tolerable body weight loss during the entire drug administration period (Figure S12A, Supporting Information). Further analyses revealed that necrosis areas were enriched in tumors co‐treated with IKE and LYG‐202, accompanied by increased levels of lipid peroxidation derivative 4‐HNE (Figure 6J,K; Figure S12B, Supporting Information). Additionally, there were no significant changes in the apoptosis marker cleaved caspase‐3 or the cell proliferation marker Ki‐67 between different groups (Figure 6L,M; Figure S12B, Supporting Information). Altogether, our work demonstrates that ALDOA inhibitors specifically increase ferroptosis susceptibility in cancer cells both in vitro and in vivo, highlighting their potential as valuable treatments for a broad array of cancers.

## 3 Discussion

In this study, we identify ALDOA as a targetable vulnerability for ferroptosis sensitization in cancer, demonstrating that its genetic or pharmacological suppression specifically enhances ferroptosis susceptibility in cancer cells. Metabolic reprogramming, notably via aerobic glycolysis‐also known as the Warburg effect‐is a hallmark of tumor initiation and progression.^[^ ^35^ ^]^ This enhanced glycolysis meets the increased bioenergetic and biosynthetic demands of highly proliferative cancer cells and supports their survival by mitigating oxidative stress.^[^ ^35^, ^36^ ^]^ Despite these insights, a direct relationship between the Warburg effect and ferroptosis resistance in cancer cells remains unclear.

Our findings provide detailed and compelling evidence that cancer cells depend on the glycolytic enzyme ALDOA to defend against ferroptosis. The elevated expression of ALDOA sustains anti‐ferroptosis lipid profile in cancer cells, thereby protecting these cells against lipid peroxidation. Mechanistically, ALDOA suppression induces metabolic reprogramming and F1,6BP accumulation, which promotes autophagy induction and ubiquitination of MBOAT2 in cancer cells. This, in turn, triggers the autophagy‐dependent degradation of MBOAT2, thereby reducing MUFA‐PL synthesis and generating a pro‐ferroptotic lipid profile (Figure S13, Supporting Information). Notably, since ACSL4 expression remains constant upon ALDOA suppression, the observed PUFA‐PL enrichment likely results from MBOAT2 downregulation‐mediated reduction in MUFA‐PL synthesis, thereby leaving more phospholipids backbones available for PUFA incorporation. In light of the above, our findings uncover a previously unrecognized glycolytic–lipid axis in which ALDOA‐mediated glycolytic flux maintains an anti‐ferroptotic lipid state by preventing the autophagy‐dependent degradation of MBOAT2, providing new mechanistic insight into how the Warburg effect confers ferroptosis resistance to cancer cells.

Notably, the accumulation of F1,6BP depends on the heightened rate of upstream glycolytic metabolism, a characteristic that underscores the distinct response of cancer cells compared to noncancerous cells. Although some normal cells, such as immune cells and stem cells, also transiently engage glycolysis to support proliferation or specialized functions, they primarily rely on alternative glycolytic enzymes rather than ALDOA.^[^ ^37^, ^38^, ^39^ ^]^ In addition, the expression of fructose‐1,6‐bisphosphatase 1 and 2 (FBP1 and FBP2), enzymes responsible for the conversion of F1,6BP to Fructose 6‐phosphate (F6P), is broadly downregulated in cancer cells,^[^ ^40^, ^41^, ^42^ ^]^ further favoring F1,6BP accumulation. Consistent with this, exogenous supplementation of F1,6BP also promoted ferroptosis sensitivity in 786‐O cancer cells, and this ferroptosis‐promoting effect was transient in wild‐type 786‐O cells due to the rapid metabolism of F1,6BP. Moreover, F1,6BP supplementation enhanced autophagy‐dependent degradation of MBOAT2 in 786‐O cells, recapitulating the effects of ALDOA depletion. Conversely, suppression of another upstream glycolytic enzyme, GPI—which inhibits downstream F1,6BP accumulation—reversed the enhanced ferroptosis induced by ALDOA depletion. These findings highlight the essential role of F1,6BP accumulation in ALDOA suppression‐mediated ferroptosis sensitization in cancer cells. Notably, since GPI inhibition, similar to ALDOA inhibition, may also block downstream glycolytic flux and redirect glucose into the pentose phosphate pathway (PPP) but exerts the opposite effect on ferroptosis regulation, this observation rules out PPP involvement in ALDOA depletion–mediated ferroptosis sensitization. Together, these data establish a mechanistic link between glycolytic reprogramming and ferroptosis regulation. In future studies, it will be valuable to extend validation to patient‐derived tumor organoids and xenograft models to further assess translational potential in more clinically relevant settings. Collectively, these results identify ALDOA‐mediated metabolic reprogramming as a targetable vulnerability for ferroptosis sensitization in cancer, paving the way for safe and effective cancer therapies through ferroptosis induction.

## 4 Conclusion

In summary, this study identifies ALDOA as a cancer‐specific metabolic vulnerability that connects glycolytic reprogramming with phospholipid remodeling to control ferroptosis sensitivity. ALDOA depletion leads to the accumulation of fructose‐1,6‐bisphosphate, which promotes autophagy activation and ubiquitination of MBOAT2, resulting in its autophagy‐dependent degradation. Loss of MBOAT2 reduces MUFA‐PL synthesis and shifts lipid remodeling toward enrichment of PUFA‐PLs, thereby generating a pro‐ferroptotic lipid landscape. Notably, these effects are preferentially observed in cancer cells, whereas noncancerous cells do not display such responses upon ALDOA depletion. Collectively, our findings establish a direct mechanistic connection between metabolic reprogramming and ferroptosis regulation, and highlight ALDOA as a potential therapeutic target for selectively sensitizing cancer cells to ferroptosis.

## 5 Experimental Section

Please refer to the Supporting Information file for methodologies and materials used in this study.

## Conflict of Interest

The authors declare no conflict of interest.

## Author Contributions

P.W. and K.H. contributed equally to this work. S.D. and P.W. conceived and guided the project with support from T.M.; P.W., S.D., and T.M. designed experiments. P.W. performed most of the experiments and analyzed the data with help from K.H., B.W., W.Z., and Y.Z. Cellular studies as well as the related biochemical studies were conducted by P.W. and K.H. with help from Y.Z. Animal experiments were performed by P.W. and B.W. Immunohistochemistry studies were conducted by P.W. and W.Z.; P.W., S.D., and T.M. wrote the manuscript.

## Supporting information



Supporting Information

## Data Availability

All data supporting the findings of this study are available within the article and its Supporting Information. Raw metabolomics and lipidomics data have been deposited in Mendeley Data (doi:10.17632/ngttmkkhkn.1). An example of the gating strategy for the lipid peroxidation assay is provided in Figure S14. Additional information is available from the corresponding author upon reasonable request.
