# Supplementary material for: ALDOA‐Mediated Metabolic Reprogramming is a Targetable Vulnerability for Ferroptosis Sensitization in Cancer
Source: Adv Sci (Weinh). 2025 Nov 11;13(6):e11880. doi: 10.1002/advs.202511880 (PMC12866770; doi:10.1002/advs.202511880)
Supplement: Supplementary file 1 — Supporting Information [file ADVS-13-e11880-s001.docx]

Supporting Information

**A****LDOA-Mediated Metabolic Reprogramming is a Targetable Vulnerability for Ferroptosis Sensitization in Cancer**

*Pengqi Wang, Kezhang He, Bowen Wang, Wei Zho**u, Ying Zhang, Tianhua Ma^*^, Sheng Ding*^*^

P. Wang, K. He, B. Wang, W. Zhou, T. Ma, S. Ding

New Cornerstone Science Laboratory, School of Pharmaceutical Sciences, Tsinghua University, Beijing, China

E-mail: matianhua@tsinghua.edu.cn; [shengding@tsinghua.edu.cn](mailto:shengding@tsinghua.edu.cn)

Y. Zhang

School of Life Sciences, Tsinghua University, Beijing, China

S. Ding

Tsinghua-Peking Joint Center for Life Sciences, Tsinghua University, Beijing, China

P. Wang and K. He contributed equally to this work

**
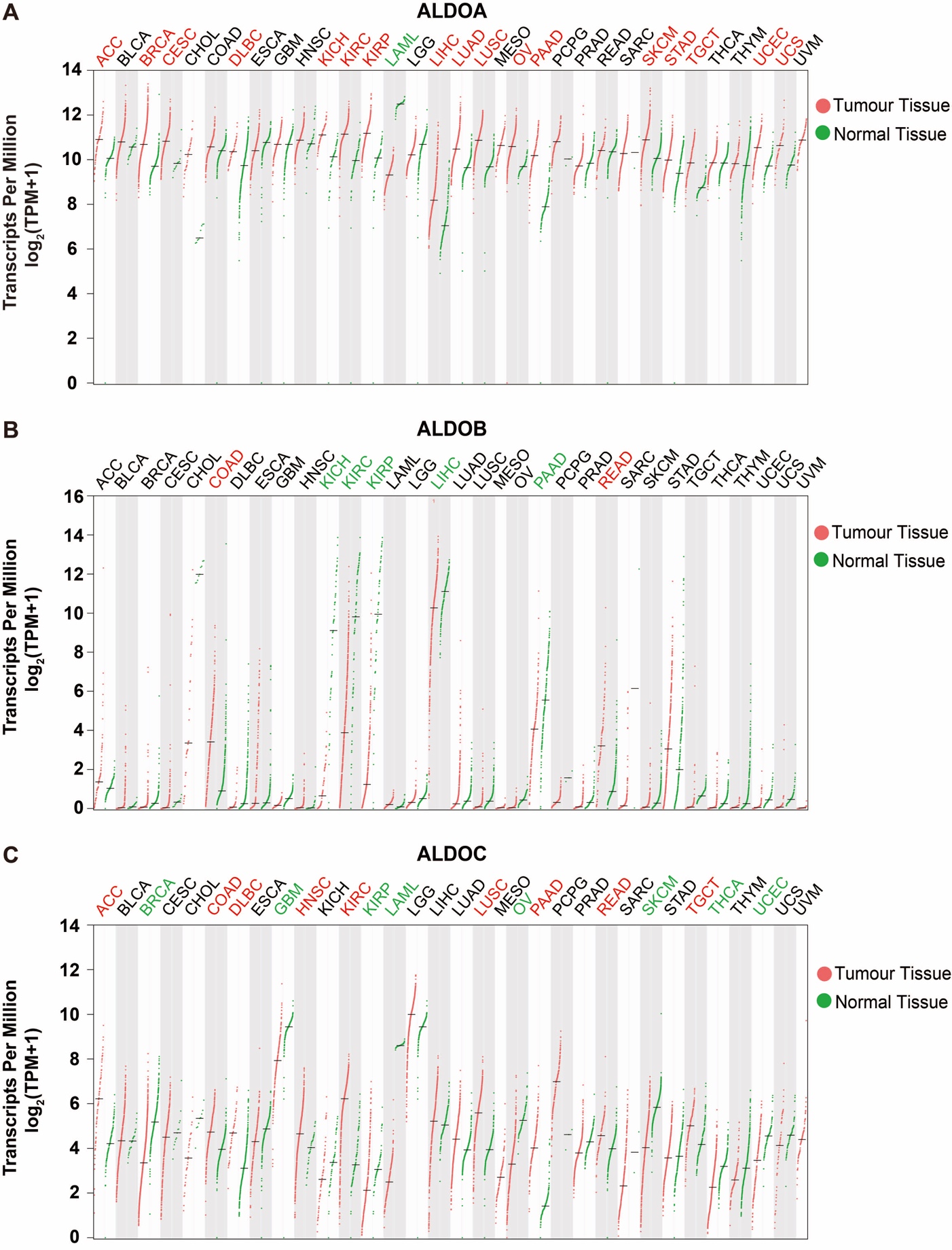
Figure S1. Comparative analysis of aldolase family gene expression in tumor and paired normal tissues**

(A-C) Gene expression profiles of *ALDOA* (A), *ALDOB* (B), and *ALDOC* (C) across TCGA tumor types with matched normal tissues assembled from TCGA and GTEx via GEPIA2; expression values are log_2_(TPM+1). ACC, Adrenocortical carcinoma; BLCA, Bladder urothelial carcinoma; BRCA, Breast invasive carcinoma; CESC, Cervical squamous cell carcinoma and endocervical adenocarcinoma; CHOL, Cholangio carcinoma; COAD, Colon adenocarcinoma; DLBC, Lymphoid neoplasm diffuse large B-cell lymphoma; ESCA, Esophageal carcinoma; GBM, Glioblastoma multiforme; HNSC, Head and neck squamous cell carcinoma; KICH, Kidney chromophobe; KIRC, Kidney renal clear cell carcinoma; KIRP, Kidney renal papillary cell carcinoma; LAML, Acute myeloid leukemia; LGG, Brain lower grade glioma; LIHC, Liver hepatocellular carcinoma; LUAD, Lung adenocarcinoma; LUSC, Lung squamous cell carcinoma; MESO, Mesothelioma; OV, Ovarian serous cystadenocarcinoma; PAAD, Pancreatic adenocarcinoma; PCPG, Pheochromocytoma and paraganglioma; PRAD, Prostate adenocarcinoma; READ, Rectum adenocarcinoma; SARC, Sarcoma; SKCM, Skin cutaneous melanoma; STAD, Stomach adenocarcinoma; TGCT, Testicular germ cell tumors; THCA, Thyroid carcinoma; THYM, Thymoma; UCEC, Uterine corpus endometrial carcinoma; UCS, Uterine carcinosarcoma; UVM, Uveal melanoma. Tumor-type labels in red indicate significantly higher expression in tumors relative to matched normal tissues, whereas green indicates significantly lower expression (criteria: FC ≥ 1.5 for upregulation or FC ≤ 0.67 for downregulation, with FDR < 0.01).

**
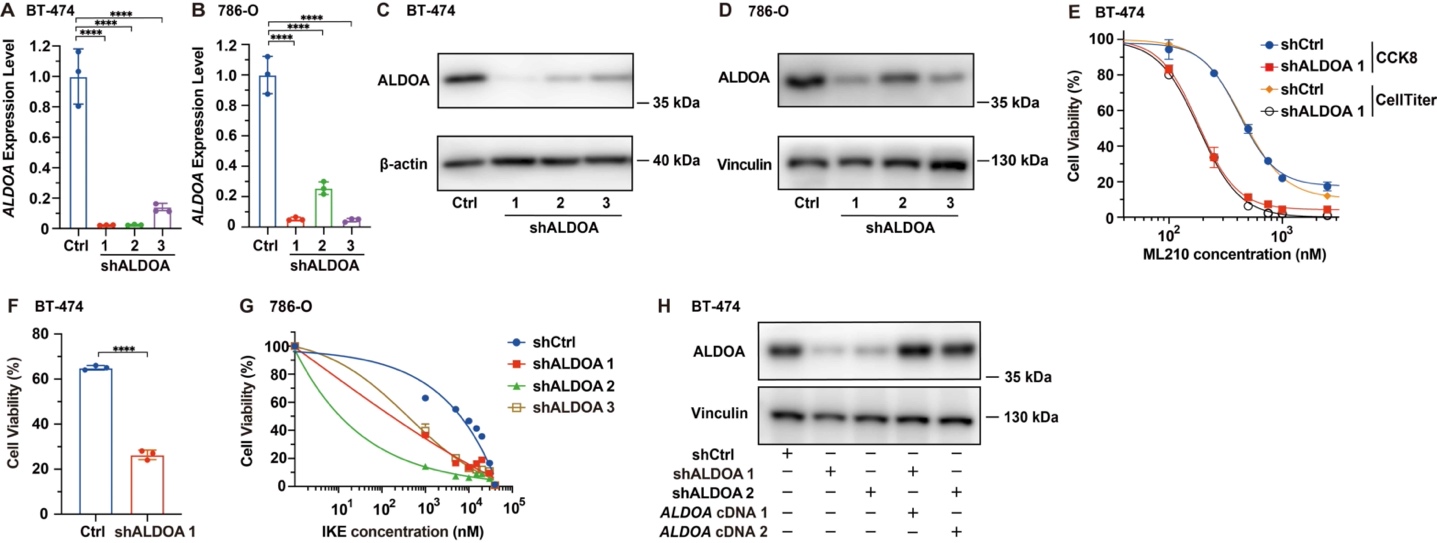
Figure S2.** **ALDOA depletion increases ferroptosis susceptibility in BT-474 and 786-O cancer cells**

(A, B) Construction of BT-474 (A) and 786-O (B) cells expressing shCtrl or shALDOA and validation of knockdown efficiency by RT-qPCR.

(C, D) Validation of ALDOA knockdown efficiency in BT-474 (C) and 786-O (D) cells expressing shCtrl or shALDOA using the Western blot analysis.

(E) Comparison of cell viability measured by CCK-8 and CellTiter-Glo assays, showing negligible differences. All subsequent viability measurements in this study were performed using CellTiter-Glo.

(F, G) Cell viability of the indicated BT-474 (F) and 786-O (G) cells treated with 25 μM IKE (F) or the indicated concentration of IKE (G) for 24 h.

(H) Re-expression of shRNA-resistant ALDOA cDNA in ALDOA-depleted BT-474 cells and validation of ALDOA restoration by Western blot analysis.

Data are mean ± s.d. of n = 3 biological replicates in (E-G). For RT-qPCR assays, data are mean ± s.d. from n = 3 technical replicates in (A,B). Data are representative of three independent experiments (A,B) or two independent experiments (C-H). Statistical analysis was performed using one-way ANOVA for (A, B) and two-tailed unpaired Student’s t-test for (F). ****P < 0.0001.

**
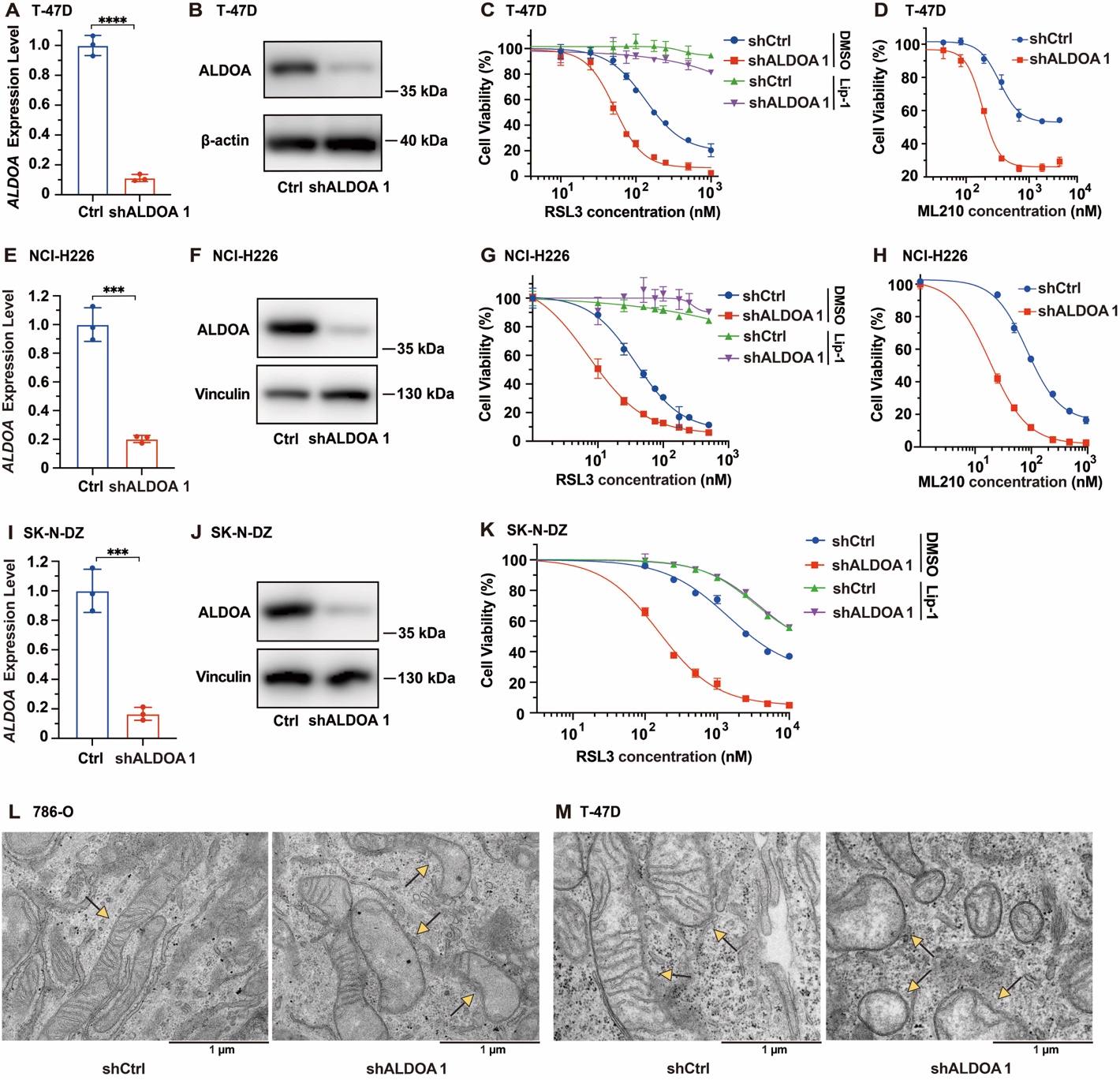
Figure S3. ALDOA depletion promotes ferroptosis sensitivity cross multiple cancer cell types**

(A, B) Construction of T-47D cells expressing shCtrl or shALDOA and validation of knockdown efficiency by RT-qPCR (A) and Western blot analysis (B).

(C) Dose-dependent toxicity of RSL3 in control and ALDOA-depleted T-47D cells in the presence or absence of 500 nM ferroptosis inhibitor Lip-1. Cell viability was assessed after 24 h of treatment.

(D) Dose-dependent toxicity of ML210 in the indicated T-47D cells. Cell viability was assessed after 24 h of treatment.

(E, F) Construction of NCI-H226 cells expressing shCtrl or shALDOA, and validation of knockdown efficiency by RT-qPCR (E) and Western blot analysis (F).

(G) Dose-dependent toxicity of RSL3 in control and ALDOA-depleted NCI-H226 cells in the presence or absence of 500 nM Lip-1. Cell viability was assessed after 24 h of treatment.

(H) Dose-dependent cytotoxicity of ML210 in the indicated NCI-H226 cells. Cell viability was assessed after 24 h of treatment.

(I, J) Construction of SK-N-DZ cells expressing shCtrl or shALDOA, and validation of knockdown efficiency by RT-qPCR (I) and Western blot analysis (J).

(K) Dose-dependent cytotoxicity of RSL3 in control and ALDOA-depleted SK-N-DZ cells in the presence or absence of 500 nM Lip-1. Cell viability was assessed after 24 h of treatment.

(L,M) Transmission electron microscopy (TEM) images showing mitochondrial morphology in the indicated 786-O (L) and T-47D (M) cells after RSL3 treatment (100 nM, 12 h for 786-O; 200 nM, 12 h for T-47D). Scale bars, 1 μm.

Data are mean ± s.d. of n = 3 biological replicates in (C,D,G,H,K). For RT-qPCR assays (A,E,I), data are mean ± s.d. of n = 3 technical replicates. All data are representative of two independent experiments. Statistical analysis was performed using two-tailed unpaired Student’s t-test in (A,E,I). ***P < 0.001, ****P < 0.0001.

**
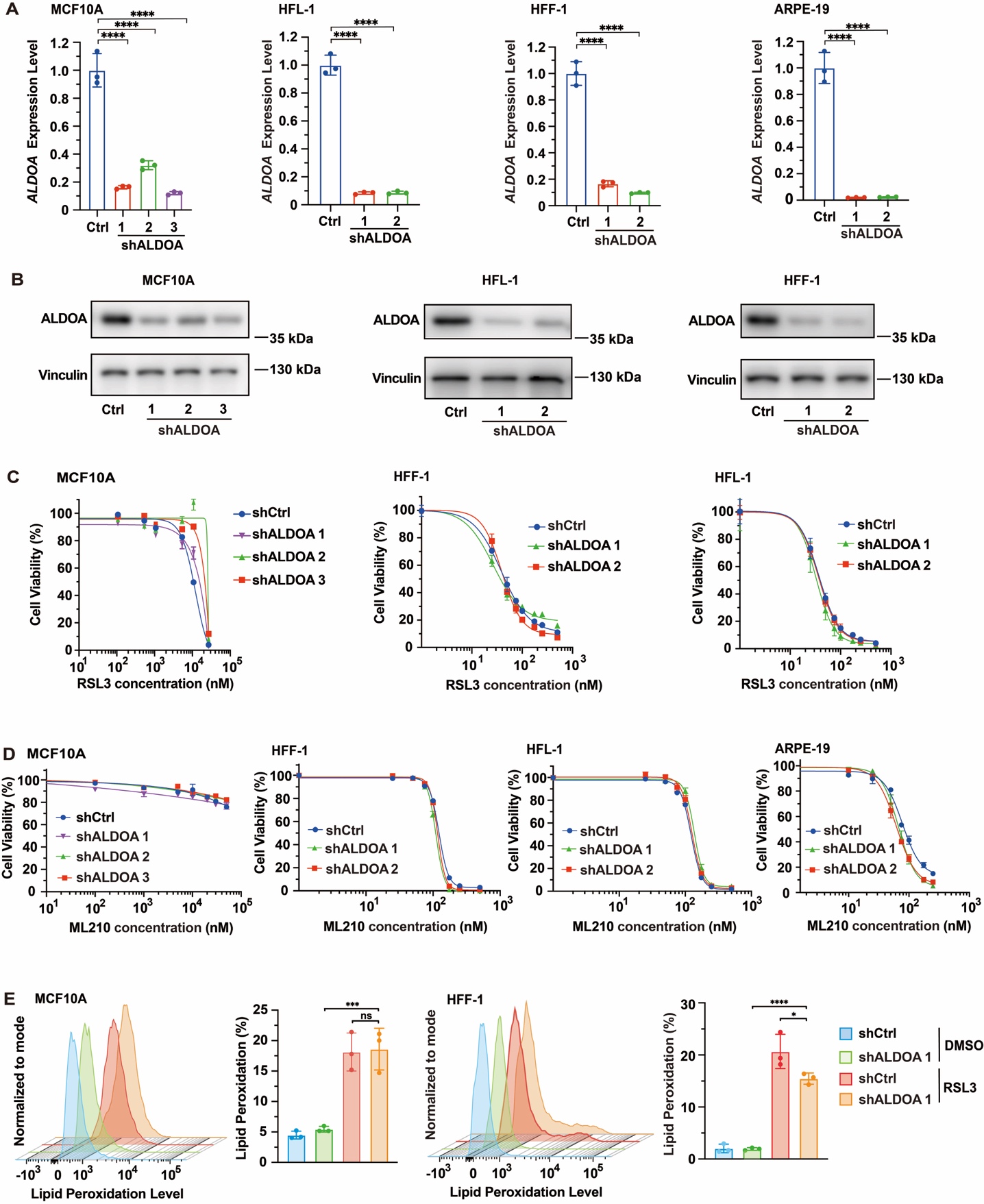
Figure S4. ALDOA has no effect on ferroptosis sensitivity in noncancerous cells**

(A) Construction of MCF10A, HFL-1, HFF-1, and APRE19 cells expressing shCtrl or shALDOA, and validation the knockdown efficiency using RT-qPCR analysis.

(B) Western blot analysis confirming ALDOA depletion in MCF10A, HFL-1, and HFF-1 cells .

(C, D) Dose-dependent cytotoxicity of RSL3 and ML210 in control and ALDOA-depleted MCF10A, HFL-1, HFF-1 and ARPE-19 cells. Cell viability was measured after treatment with RSL3 or ML210 for 24 h.

(E) Lipid peroxidation assessment of indicated MCF10A cells treated with 20 μM RSL3 for 12 h, and of indicated HFF-1 cells treated with 50 nM RSL3 for 8 h.

Data are mean ± s.d. of n = 3 biological replicates in (C-E) or n=3 technical replicates in (A). Data are representative of two independent experiments (A-E). Statistical analysis was performed using one-way analysis of variance (ANOVA) in (A) and two-way ANOVA in (E). *P < 0.05, ***P < 0.001, ****P < 0.0001. ns, not significant.


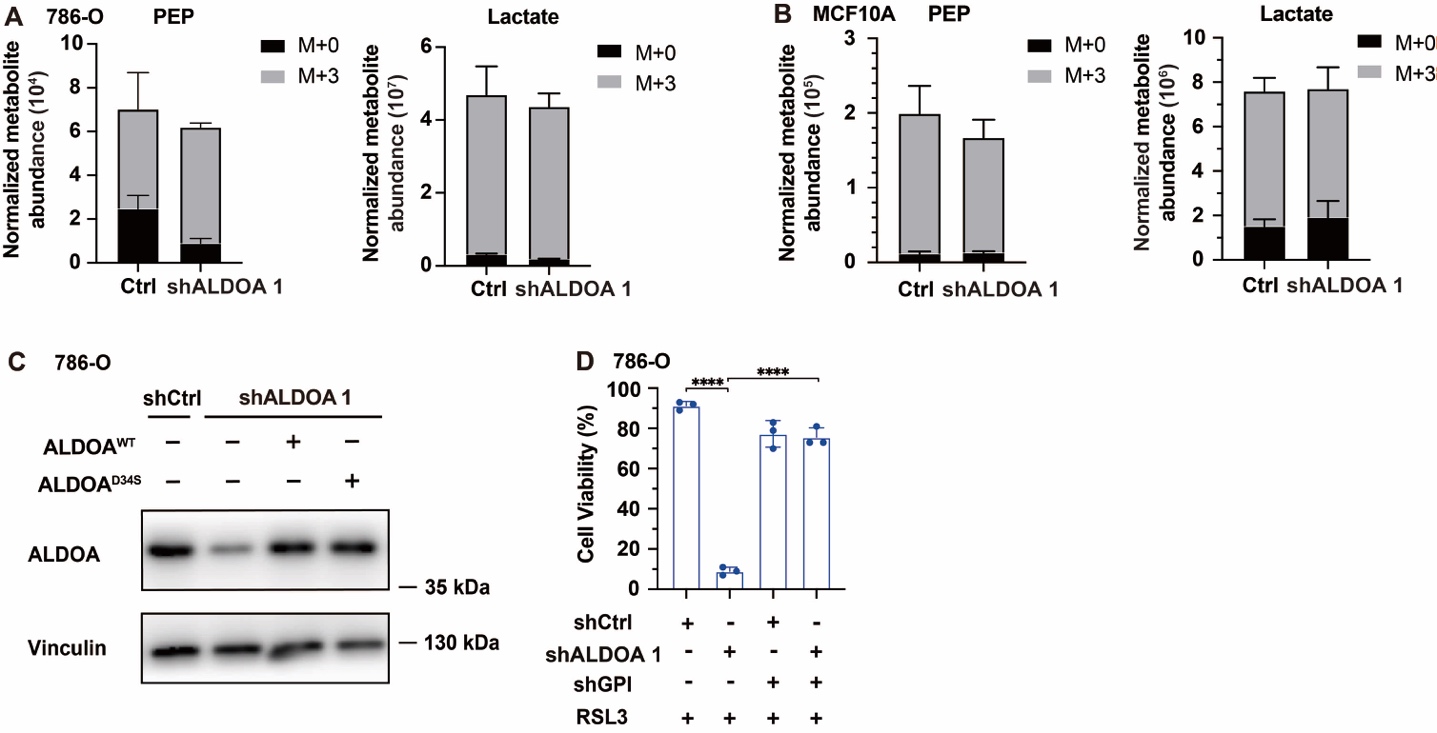
**Figure S5.** **ALDOA depletion elicits a divergent metabolic response in cancer versus noncancerous cells**

(A, B) Labeled and unlabeled metabolite levels for phosphoenolpyruvate (PEP) and lactate in 786O (A) and MCF10A (B) cells expressing either shCtrl or shALDOA. Cells were cultured in full medium or glucose-deficient medium containing U-^13^C-glucose for 0.5 h. Natural isotope corrected isotopologue abundances normalized to biomass are shown.

(C) Western blot analysis showing the expression of ALDOA in 786-O cells expressing shCtrl, shALDOA, or shALDOA complemented with WT or D34S ALDOA.

(D) Cell viability of 786-O cells under control and ALDOA-depleted conditions, with or without GPI depletion, following treatment with 50 nM RSL3 for 24 h.

Data are mean ± s.d. of n = 3 biological replicates in (A, B, D). Data are representative of two independent experiments (C, D) or one experiment (A, B). Statistical analysis was performed using two-tailed unpaired Student’s t-test in (D). ****P < 0.0001.

**
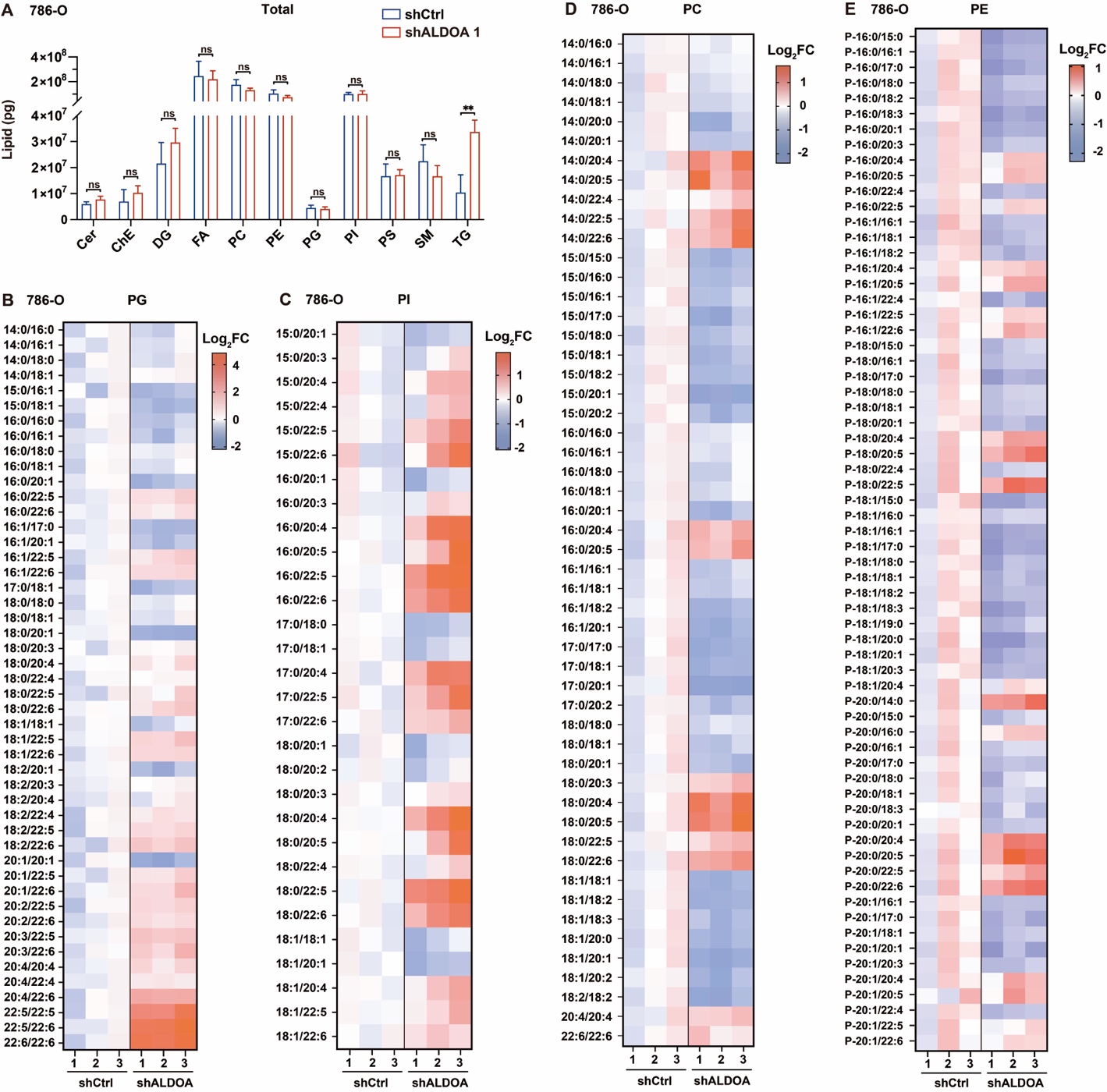
Figure S6. ALDOA regulates ferroptosis through phospholipid remodeling**

(A) Quantification of the content of total lipids in control and ALDOA-depleted 786-O cells by high throughput lipidomic analysis. Y axis indicates the normalized weight of lipid (pg) per 6 x10^6^ cells.

(B-E) Heat map showing the normalized abundance of phosphatidylglycerol (PG) (B), phosphatidylinositol (PI) (C), phosphatidylcholine (PC) (D) and phosphatidylethanolamine (PE) (E) in control and ALDOA-depleted 786-O cells by high throughput lipidomic analysis.

Data are mean ± s.d. of n = 3 biological replicates from one independent experiment in (A-E). Statistical analysis was performed using two-tailed unpaired Student’s t-test in (A). **P < 0.01. ns, not significant.


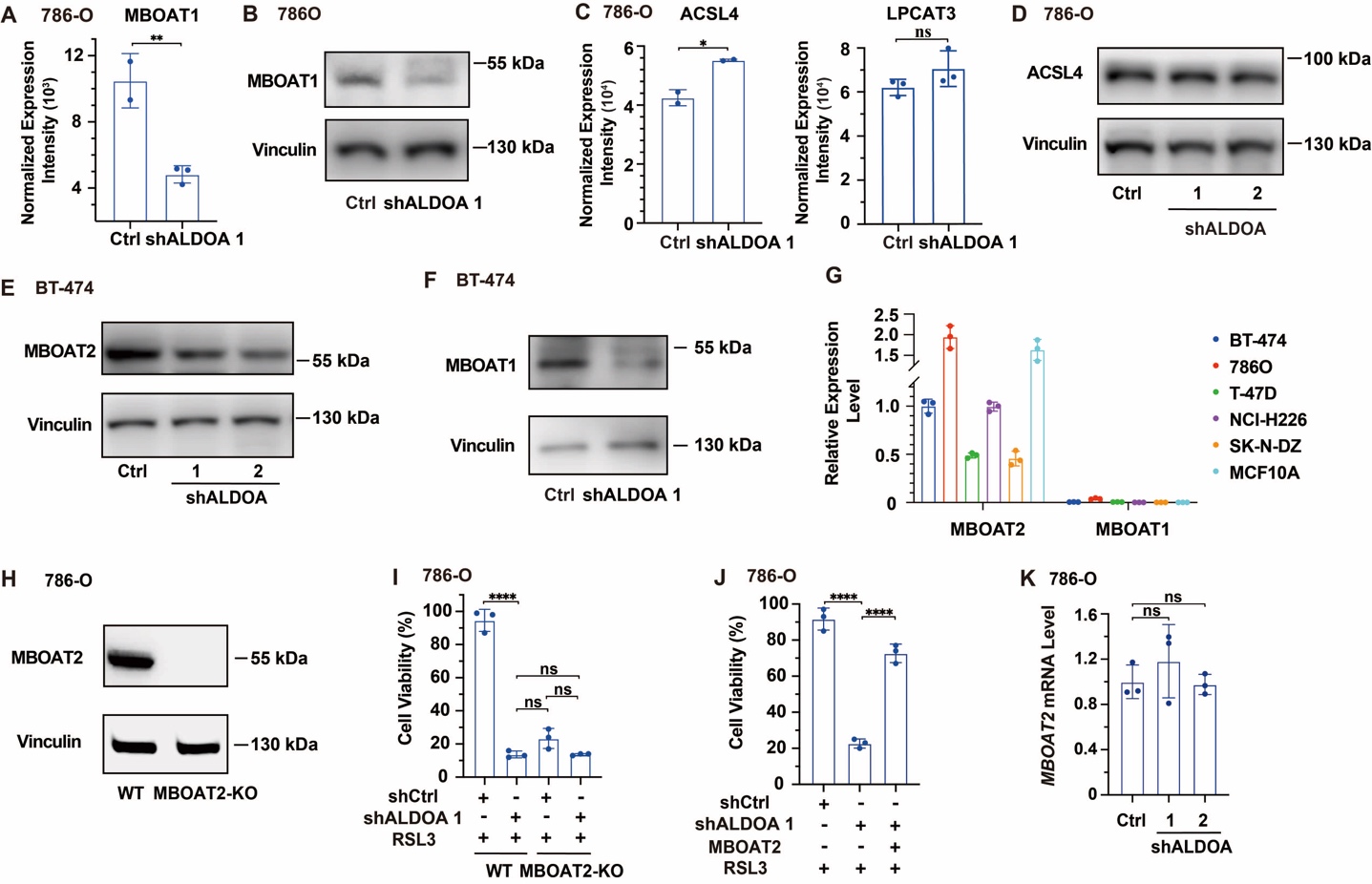
**Figure S7. ALDOA depletion reduces MBOAT2 protein levels to drive phospholipid remodeling and ferroptosis sensitization in cancer cells**

(A) Proteomic quantification of MBOAT1 protein levels in control and ALDOA-depleted 786-O cells.

(B) Western blot analysis of MBOAT1 protein levels in the indicated 786-O cells.

(C) Proteomic quantification of ACSL4 and LPCAT3 in the indicated 786-O cells (note: some ACSL4 data points are missing in the proteomic data).

(D) Western blot analysis of ACSL4 protein levels in the indicated 786-O cells.

(E, F) Western blot analysis of MBOAT2 (E) and MBOAT1 (F) protein levels in control and ALDOA-depleted BT-474 cells.

(G) Relative expression levels of MBOAT2 and MBOAT1 in the indicated cells lines, as determined by RT–qPCR.

(H) Western blot analysis of MBOAT2 protein levels in MBOAT2 knockout 786-O cells.

(I) Cell viability of wild-type and MBOAT2-KO 786-O cells expressing shCtrl or shALDOA after treatment with 50 nM RSL3 for 24 h.

(J) Viability of the 786-O cells expressing shCtrl, shALDOA, or a combination of shALDOA and MBOAT2, following treatment with 50 nM RSL3 for 24 h.

(K) MBOAT2 mRNA levels in control and ALDOA-depleted 786-O cells determined by RT–qPCR.

Data are mean ± s.d. of n = 3 biological replicates in (A, C, I, J). For RT-qPCR assays, data are mean ± s.d. of n = 3 technical replicates in (G, K). Data are representative of one (A, C) or two (B, D-K) independent experiments. Statistical analysis was performed using two-tailed unpaired Student’s t-test in (A, C, I-K). *P < 0.05, **P < 0.01, ****P < 0.0001. ns, not significant.


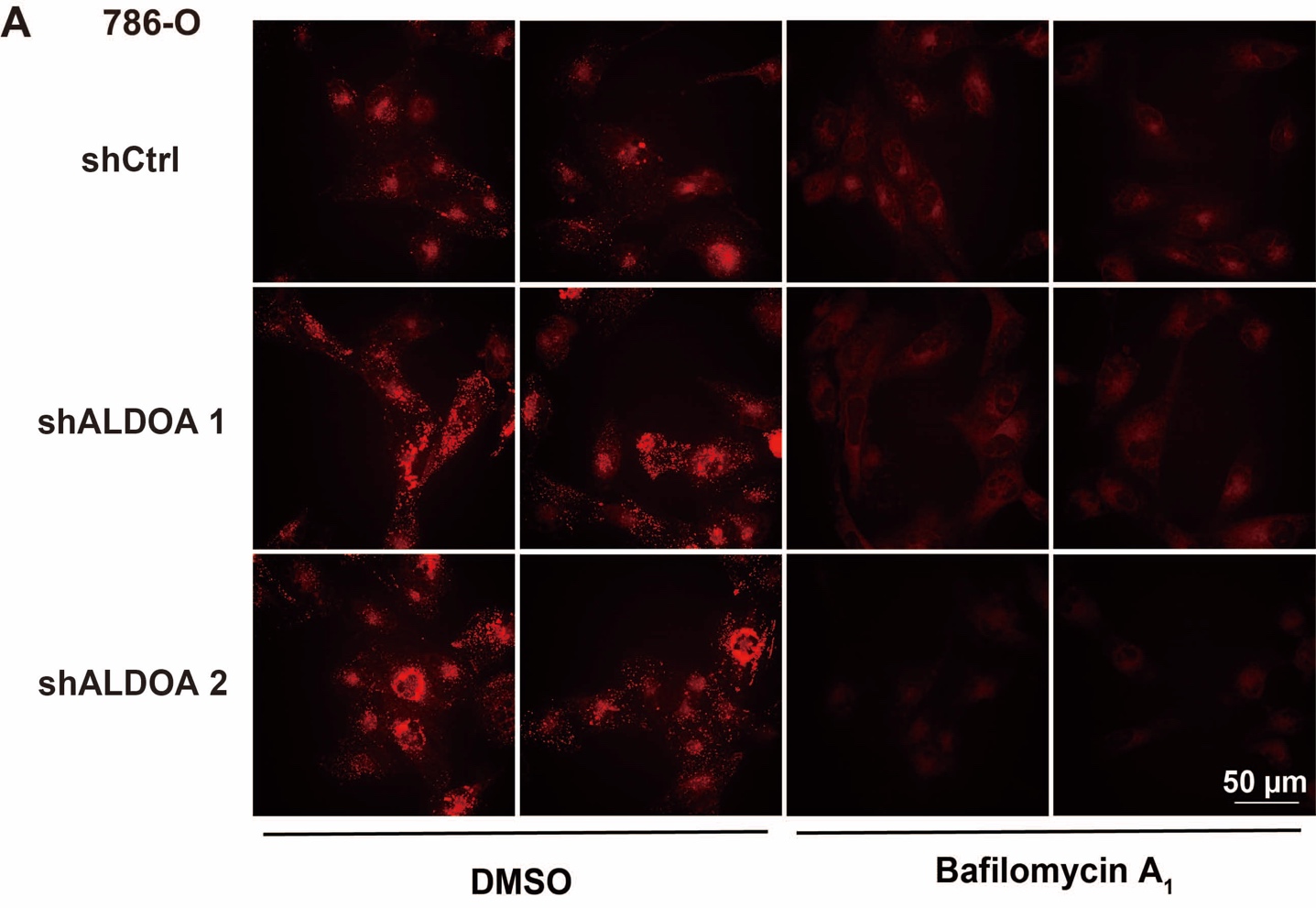
**Figure S8.** **ALDOA depletion activates autophagy in cancer cells**

(A) LysoTracker staining in control and ALDOA-depleted 786-O cells with or without pretreatment with 100 nM bafilomycin A1 for 24 h. Scale bar, 50 μm. Data are representative of three independent experiments.


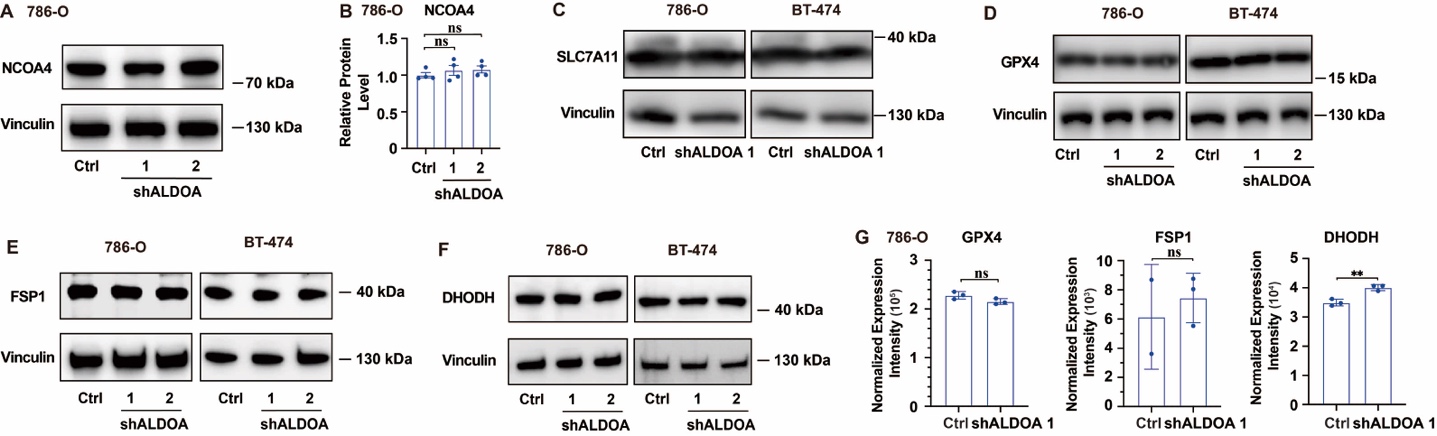
**Figure S9. ALDOA depletion does not affect other canonical ferroptosis pathways**

(A, B) Western blot (A) and quantification (B) of NCOA4 protein levels in control and ALDOA- depleted 786-O cells.

(C-F) Western blot analysis of SLC7A11 (C), GPX4 (D), FSP1 (E) and DHODH (F) protein levels in control and ALDOA-depleted 786-O and BT-474 cells.

(G) Proteomic analysis of GPX4, FSP1 (AIFM2) and DHODH protein levels in the indicated 786-O cells. One data point for FSP1 is missing in the proteomic data.

Data are mean ± s.d. of n = 4 biological replicates in (B) and n = 3 biological replicates in (G). Data are representative of three (A,B), two (C-F) or one (G) independent experiments. Statistical analysis was performed using two-tailed unpaired Student’s t-test in (B,G). **P < 0.01. ns, not significant.

**
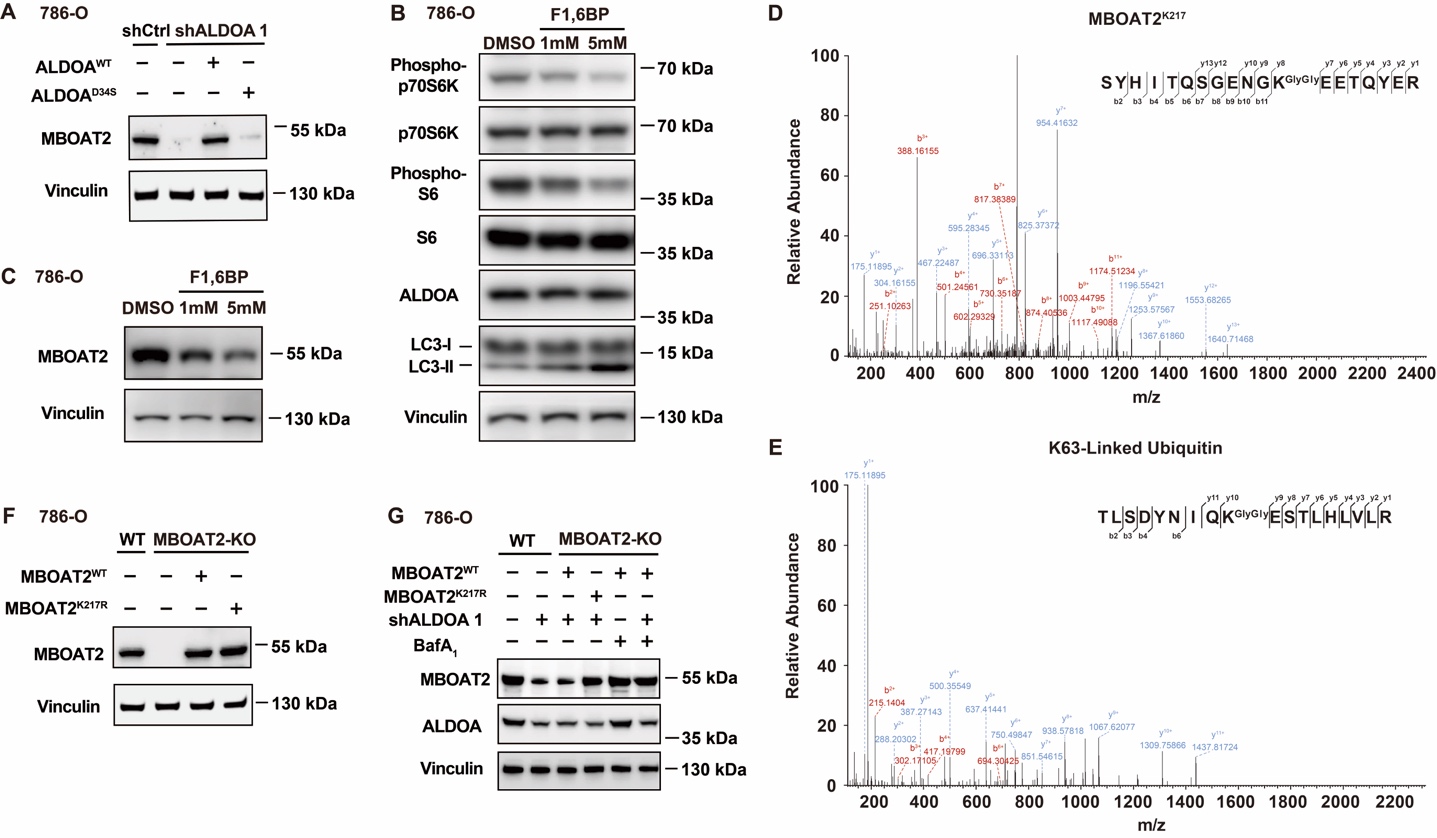
Figure S10. ALDOA-depletion-induced F1,6BP accumulation promotes ubiquitination-dependent autophagic degradation of MBOAT2.**

(A) Western blot analysis of MBOAT2 protein levels in 786-O cells expressing shCtrl, shALDOA, or shALDOA complemented with wild-type (WT) or catalytically inactive D34S ALDOA.

(B) Detection of mTORC1 activity (phosphorylation of S6 and p70S6K) and autophagy (LC3 turnover) in 786-O cells treated with or without the indicated concentrations of F1,6BP. ALDOA expression was also analyzed in these cells.
(C) Western blot analysis of MBOAT2 protein levels in 786-O cells treated with 1 or 5 mM F1,6BP for 24 h.
(D) Representative LC–MS/MS spectrum identifying lysine 217 (K217) of MBOAT2 as the ubiquitination site.
(E) Representative LC–MS/MS spectrum confirming the presence of K63-linked polyubiquitin chains.
(F) Western blot analysis of MBOAT2 protein levels in WT, MBOAT2-KO, and MBOAT2-KO 786-O cells re-expressing WT or K217R mutant MBOAT2.
(G) Western blot analysis of MBOAT2 and ALDOA in WT, MBOAT2-KO, and MBOAT2-KO 786-O cells re-expressing WT or K217R MBOAT2, with or without ALDOA depletion. Bafilomycin A1 treatment was applied to inhibit autophagy-dependent degradation of MBOAT2.

Data are representative of three (B,C) or two (A,D-G) independent experiments.


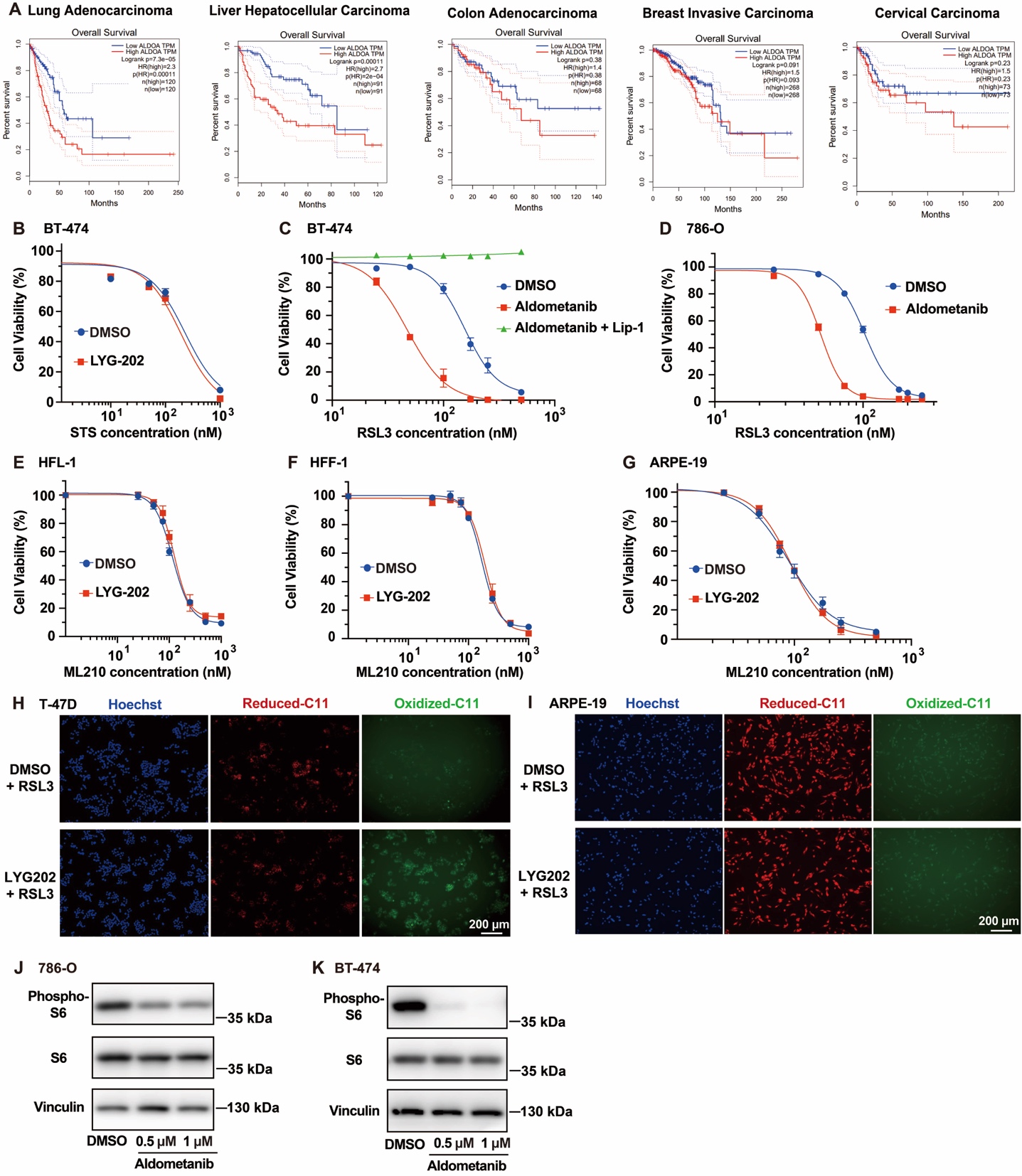
**Figure S11.** **ALDOA inhibitors promote ferroptosis in cancer cells but not in non-cancerous cells**

(A) Lower ALDOA expression correlated with improved prognosis, although colon, breast, and cervical cancers showed the same trend without statistical significance (p > 0.05), likely due to limited sample size.

(B) Viability of the BT-474 cells under the treatment of the indicated concentrations of staurosporine (STS) for 24 h, with or without pretreatment with 4 μM LYG-202 for 24 h.

(C, D) Viability of the BT-474 (C) and 786-O (D) cells treated with the indicated concentrations of RSL3 for 24 h, with or without pretreatment with 500 nM (C) or 1 μM (D) Aldometanib for 12h. Lip-1 (500nM) was used as a ferroptosis inhibitor (C).

(E-G) Viability of HFL-1 (E), HFF-1 (F), and ARPE-19 (G) cells treated with the indicated concentrations of ML210 for 24 h, with or without pretreatment with 4 μM LYG-202 for 24h.

(H, I) Lipid peroxidation in T-47D (H) and APRE19 (I) cells assessed by C11-BODIPY 581/591 staining. Cells were pretreated with 4 μM LYG-202 for 24 h, followed by treatment with 1 μM (H) or 500 nM (I) RSL3 for 12h. Fluorescent images were acquired at 488 nm (oxidized form) and 563 nm (reduced form). Scale bar, 200 μm.

(J, K) mTORC1 activity indicated by the phosphorylation of ribosome S6 (S6) in 786-O and BT-474 cells treated with the indicated concentration of Aldometanib for 24 h.

Data are mean ± s.d. of n = 3 biological replicates in (B-G). Data are representative of three independent experiments (B-D) or two independent experiments (E-K).

**
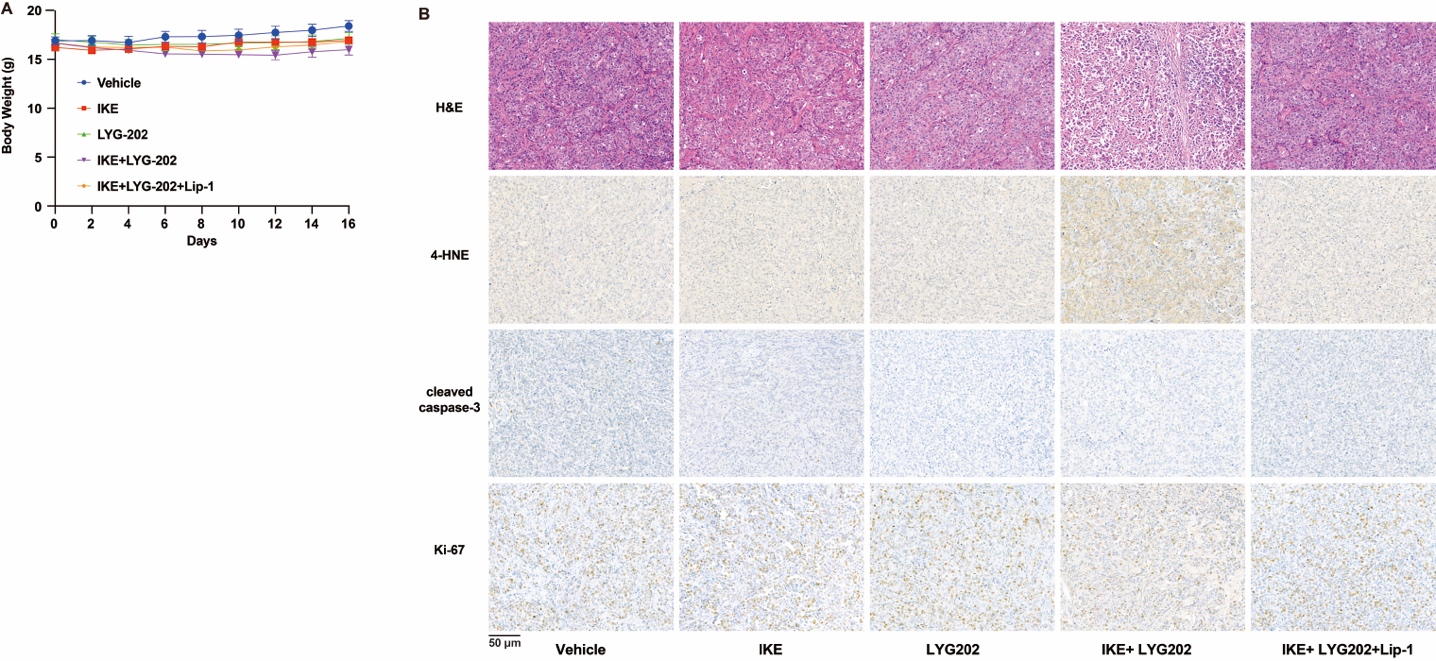
****Figure S12. ALDOA inhibitor LYG-202 promotes ferroptosis in cancer xenografts**

(A) Body weight of BT-474 cells xenografted mice with the indicated treatments over time. n = 6 in IKE+LYG-202 group and n = 5 in the other four groups. Data plotted are mean ± s.e.m.

(B) Representative H&E staining, as well as immunohistochemical staining of 4-HNE, cleaved caspase-3 and Ki-67 in paraffin section of BT-474 xenografts with indicated treatments. Scale bar, 50 μm.

**
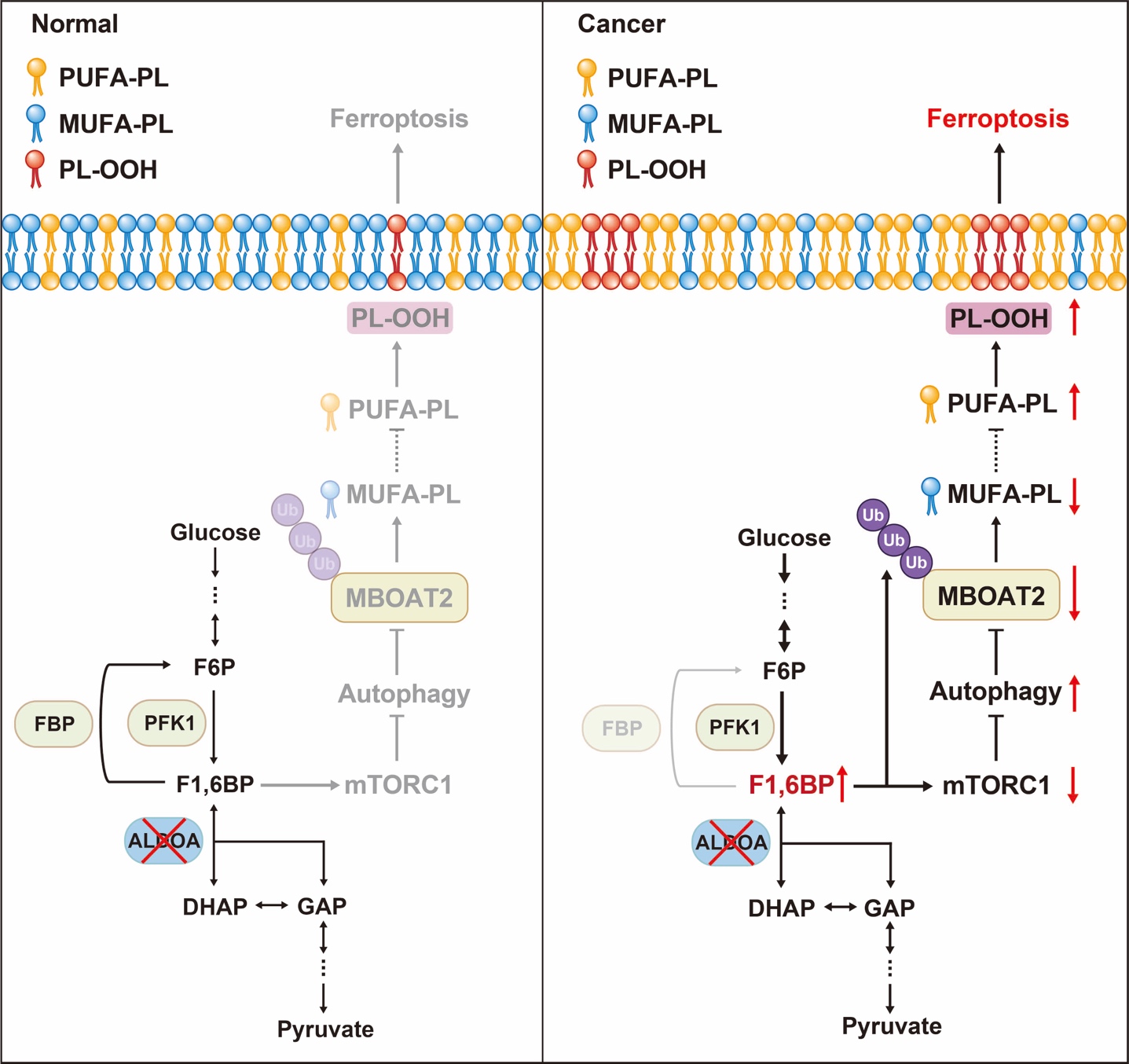
Figure S13.** **Schematic depicting how ALDOA suppression enhances ferroptosis susceptibility in cancer**

PL-OOH, phospholipid hydroperoxide; PUFA-PL, phospholipid containing polyunsaturated fatty acids; MUFA-PL, phospholipid containing monounsaturated fatty acids; MBOAT2, membrane-bound O-acyltransferase domain–containing 2; Ub, ubiquitin. Black lines indicate regulatory networks that are well-established or validated in this study, whereas gray lines denote inactive regulatory networks under the indicated conditions. Thicker glycolytic lines denote enhanced metabolic flux.

**
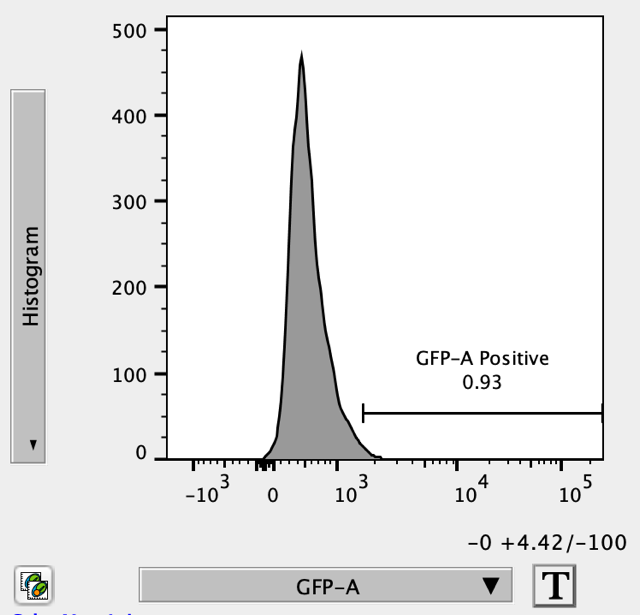

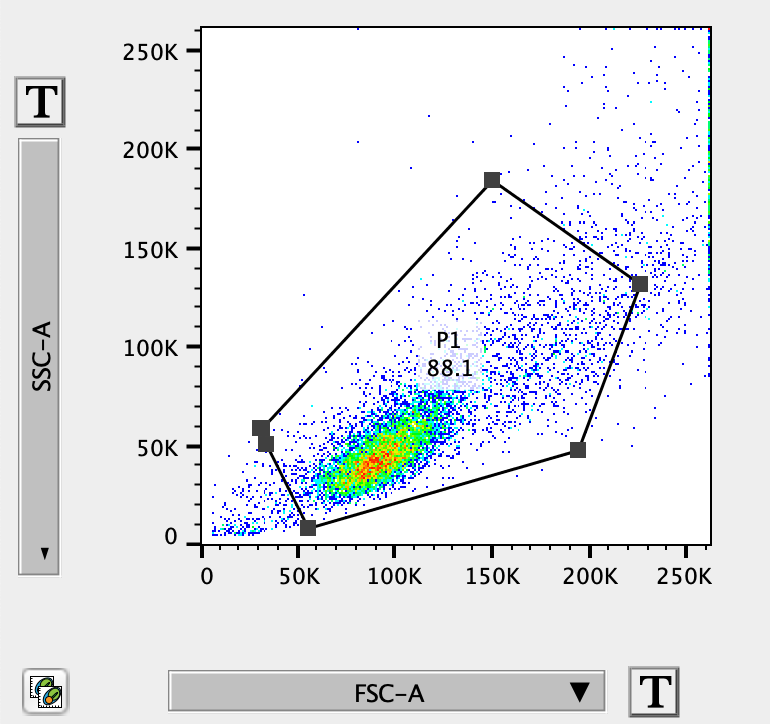
Figure S14. An example of the gating strategy for lipid peroxidation assay**
P1 area was considered as viable cells and analyzed (Left); Percentage of GFP positive cells was considered as value of lipid peroxidation (%) shown in this study (right).

**Experimental Methods**

**Cell Lines and Cell Culture**

The cell lines used in this study are commonly used in cancer research and serve as representative models of diverse tissue lineages. Human breast carcinoma cells BT-474 (RRID:CVCL_0179) and T-47D (RRID:CVCL_0553), human renal cell carcinoma cells 786-O (RRID:CVCL_1051), human pleural mesothelioma cells NCI-H226 (RRID:CVCL_1544), human embryonic kidney 293 (HEK293T; RRID:CVCL_0063) cells, human foreskin fibroblast cells HFF-1 (RRID:CVCL_3285), human lung fibroblast cells HFL-1 (RRID:CVCL_0298), human retinal pigmented epithelium cells ARPE-19 (RRID:CVCL_0145) and human mammary epithelial cells MCF10A (RRID:CVCL_0598) were obtained from the Cell Bank of the Chinese Academy of Sciences (Shanghai, China). Human neuroblastoma cells SK-N-DZ (RRID:CVCL_1701) were originally obtained from the American Type Culture Collection (ATCC). All cell lines were routinely tested and confirmed to be free of mycoplasma and other potential contaminants. Short tandem repeat (STR) profiling was performed to authenticate their identities.

BT-474, 786-O, and NCI-H226 cell lines were cultured in RPMI 1640 (Gibco) with 10% FBS (Gibco) and 1% penicillin–streptomycin. T-47D, SK-N-DZ, HEK293T and HFF-1 cells were cultured in DMEM (Gibco) with 10% FBS (Gibco) and 1% penicillin–streptomycin. HFL-1 cells were cultured in F-12K (Gibco) with 10% FBS (Gibco) and 1% penicillin–streptomycin. ARPE-19 cells were cultured in DMEM/F12(1:1) (Gibco) with 10% FBS (Gibco) and 1% penicillin–streptomycin. MCF10A cells were cultured in human mammary epithelial cell basal medium (Invitrogen) with MEGS (Invitrogen). All cells were cultured in a 37°C humidified incubator under 5% CO_2_.

**Chemicals**

RSL3 (HY-100218A), ML210 (HY-100003), Staurosporine (HY-15141), Deferoxamine mesylate (HY-B0988), Necrostatin-1 (HY-15760), PRGL493 (HY-139180), Chloroquine (HY-17589A), Bafilomycin A1 (HY-100558), Aldometanib (HY-148189) and PR-619 (HY-13814) were purchased from MedChemExpress. IKE (S8877), Liproxstatin-1 (S7699), and Z-VAD-FMK (S7023) were purchased from Selleck. N-ethylmaleimide (E1271) was purchased from Sigma. LYG-202 (GC40865) and D-Fructose-1,6-bisphosphate (GC43430) were purchased from GLPBIO.

**Screening for targetable ferroptosis vulnerabilities in cancer**

The potential relationship between glycolytic enzymes and ferroptosis was explored using the Connectivity Map (CMap) online tool (https://clue.io/). Detailed analytical procedures are available in the official guidelines of this database. In brief, gene expression profiles from established ferroptosis-inducing strategies were selected from the Touchstone dataset and defined as ferroptosis reference gene expression signatures. These included treatments with erastin and sorafenib, as well as *GPX4* knockdown in all available cancer cell lines in the Touchstone dataset. Subsequently, the gene expression profiles from the knockdown of core glycolytic enzymes were compared against profiles from the ferroptosis-inducing treatments using the Touchstone tool available on the CLUE platform. Next, data were collected and analyzed to determine connectivity scores, indicating the degree of similarity between the reference and query signatures. Bar plots illustrating the results were generated using GraphPad Prism 9.

**Cell viability analysis**

Viable cells were measured by CellTiter-Glo (Beyotime, C0068XL) using a luminescent plate reader according to the manufacturer’s instructions. In brief, 3,000-8,000 cells per well were seeded into a 96-well plate (depending on cell size). After 24 h, cells were treated with different reagents at the indicated concentration for 24-72 h (as indicated in the figures or figure legends), with three independent biological replicates precondition. At the end of the treatment, cellular ATP levels were measured by CellTiter-Glo luminescent cell viability reagent following the manufacturer’s instructions on a multi-plate reader. Relative viability was normalized to the corresponding vehicle-treated (control) group unless otherwise indicated. Nonlinear regression models were used to simplify curve fitting using GraphPad Prism 9.

**Lipid peroxidation measurement**

**Flow cytometry analysis.** Cells were seeded on 12-well plates, and treated with indicated concentrations of compounds for the indicated durations (as shown in the figures or figure legends) on the following day. Then, cells were trypsinized (Invitrogen) and resuspended in 200 μl DPBS containing 5 μM C11-BODIPY 581/591 (Invitrogen, D3861). Cells were incubated at 37 °C for 30 min. Lipid peroxidation was assessed using the flow cytometer BD FACS Aria II with a 488 nm laser on an FL1 detector. Initial cell population gating (FSC and SSC) was drawn to exclude debris, and the signal from oxidized C11 was monitored and calculated as shown in Figure S14. A minimum of 1 × 10^4^ single cells were analyzed for each sample. Data were collected and analyzed using the FACS Diva software (BD, version 8.0) and FlowJo 10 software.

**BODIPY-C11 imaging.** Cells were seeded on 6-well plates the day before staining. On the following day, cells were treated with indicated concentration of chemicals for the indicated durations (as shown in the figures or figure legends). Then, cells were treated with 5 μM C11-BODIPY 581/591 and incubated at 37 °C for 30 min. Images were acquired on an Olympus IX83 inverted fluorescence microscope at 488 nm for the oxidized form BODIPY-C11 at 563 nm for the reduced form.

**Generation of cell lines with candidate gene depletion**

For the construction of ALDOA-depleted cancer and non-cancerous cell lines, shRNA plasmids targeting ALDOA (Sigma) were co-transfected into HEK293T cells together with the lentiviral packaging vectors psPAX2 (addgene, 12260) and pMD2.G (addgene, 12259) using the Lipofectamine 3000 reagent (Invitrogen, L3000075). The cell culture supernatants containing viral particles were collected 48h after transfection and filtered with 0.45 μm low protein binding syringe filter (Millipore). Target cells (e.g., BT-474, 786-O and MCF10A) were transduced with the lentiviral supernatants and, 24 h later, selected with puromycin (1–3 µg/mL; dose optimized for each cell line) and expanded for analysis. To capture the primary metabolic effects of ALDOA depletion, newly constructed ALDOA-depleted cell lines without prolonged passaging were used for downstream analyses. The construction of cell lines with the silencing of other glycolytic genes followed the same protocol. The TRC numbers of shRNAs used were as follows: shCtrl: SHC002; shALDOA #1, TRCN0000052506; shALDOA #2, TRCN0000052507; shALDOA #3, TRCN0000052503; shGPI, TRCN0000049151; shGAPDH, TRCN0000025836; shPGK1, TRCN0000195247. For construction of MBOAT2-knockout cell lines, 786-O cells were transfected with PX458 (Addgene,48138) harboring an sgRNA targeting human MBOAT2 (5′-CAGCTGCACGGCGTTGCTGA-3′) following the manufacturer’s instructions. At 48 h post-transfection, single GFP-positive cells were isolated by FACS directly into 96-well plates to establish monoclonal lines. Clones were expanded and validated by Western blotting for loss of MBOAT2 protein.

**Overexpression plasmid construct and cell line generation**

For construction of ALDOA rescue/overexpression plasmids, cDNAs encoding ALDOA-WT, the catalytically impaired ALDOA-D34S mutant, and shRNA-resistant ALDOA variants were cloned into a lentiviral expression vector (Addgene, 17446). Transfer plasmids (empty vector or ALDOA constructs) were co-transfected with psPAX2 (Addgene, 12260) and pMD2.G (Addgene, 12259) into HEK293T cells using Lipofectamine 3000. For construction of MBOAT2-WT or MBOAT2-K217R stable overexpression lines, a codon-optimized human MBOAT2 coding sequence (CDS) was inserted into the lentiviral expression vector (Addgene, 17446). Lentivirus was produced and used to transduce 786-O cells as described above. Expression of the indicated genes was confirmed by Western blotting.

**Transmission electron microscopy**

Transmission electron microscopy was performed with technical support from the Center of Biomedical Analysis, Tsinghua University. Briefly, RSL3-treated 786-O and T-47D cells were fixed in a mixture of paraformaldehyde (2%) and glutaraldehyde (2.5%) and then washed 4 times with PB buffer. Cells were post-fixed in 1% osmium tetroxide (OsO₄) containing 1.5% tetrapotassium hexacyanoferrate trihydrate for 1 h at 23°C, followed by graded ethanol dehydration (50%, 70%, 80%, 90%, 100% × 3; 2 min each). Samples were then graded-infiltrated with mixtures of ethanol and Epon 812 resin, followed by two changes of 100% Epon 812 and polymerization at 60 °C. Polymerized blocks were sectioned on a Leica EM UC7 ultramicrotome. Ultrathin sections (~70 nm) were collected on coated copper grids, contrasted on-grid with 2% uranyl acetate (25 min) and lead citrate (5 min), and imaged on a Hitachi HT7800 transmission electron microscope (80 kV, Tokyo, Japan).

**RNA extraction, cDNA synthesis, and RT-qPCR**

RNA isolation from cell pellets was performed using AxyPrep™ total RNA kit (Axygen, AP-MN-MS-RNA-250), according to the manufacturer’s instructions. For cDNA synthesis, 1 μg of total RNA from each sample was reverse transcribed into cDNA using the EasyScript™ cDNA Synthesis SuperMix (TransGen Biotech, AE341-02), following the manufacturer’s instructions. Subsequent RT-qPCR analyses were performed using PerfectStart™ qPCR SuperMix (TransGen biotech, AQ601-01-V2) on a CFX96 Real-Time PCR Detection System (Bio-Rad). The Ct values obtained were normalized against *ACTB* as a housekeeping gene using the 2^-∆∆Ct^ method. For qPCR assays, 3 technical replicates were used.

The following forward and reverse qPCR primers were used: *ALDOA*-forward: 5’-CAGGGACAAATGGCGAGACTA-3’, *ALDOA*-reverse: 5’-GGGGTGTGTTCCCCAATCTT-3’; *MBOAT2*-forward: 5’-CTCGCTGGGACTTAATTTCCAA-3’, *MBOAT2*-reverse: 5’-GGTTCGTTCATAACACACCCTT-3’; *MBOAT1*-forward: 5’-GTTTCGCATCTACTTACGTCCTG-3’, *MBOAT1*-reverse: 5’-GCACATTAACACCAGCACAAAA-3’.

**Western blotting**

Cells were lysed in RIPA buffer (Beyotime, P0013B) supplemented with protease inhibitor cocktail (Beyotime, P1006) and phosphatase inhibitor (Beyotime, P1081), and incubated on ice for 10 min. Lysate was centrifuged at 15,000g, 4 °C for 15 min. The supernatant was collected, diluted with 5 × protein loading buffer (Yeasen, 20315ES20) and incubated at 95 °C for 10 min. An equal amount of protein was loaded in each lane of 8-12% SDS-PAGE gel with PageRuler™ Prestained Protein Ladder (10-180kDa; Thermo Fisher Scientific, 26616) and transferred onto PVDF membranes (Millipore) using a Bio-Rad Western blot transfer system. Membranes were blocked with QuickBlock™ Western Blocking Buffer (Beyotime, P0252) for 1 h at room temperature and incubated with primary antibody diluted with Primary Antibody Dilution Buffer (Beyotime, P0256) overnight at 4 °C. The next day, membranes were washed in TBS containing 0.1% Tween-20 (TBST) for four times and then were incubated with HRP-conjugated secondary antibodies diluted with TBST for 1 h at room temperature. Finally, membranes were washed for 4 times with TBST and imaged on Amersham Imager 600 (GE Healthcare Life Sciences).

The following antibodies were used: anti-AldolaseA (1:1,000, 8060, CST), anti-GPX4 (1:2,000, ab125066, Abcam), anti-SLC7A11 (1:1,000, 12691S, CST), anti-ACSL4 (1:2,000, 22401-1-AP, Proteintech); anti-S6 Ribosomal Protein (1:1,000, 2217T, CST), anti-Phospho-S6 Ribosomal Protein (Ser235/236) (1:2,000, 4858T, CST), anti-p70 S6 Kinase (1:1,000, 2708, CST), anti-Phospho-p70 S6 Kinase (Thr389) (1:1,000, 9234, CST), anti-p62 (1:1,000, 18420-1-AP, Proteintech), anti-LC3B (1:1,000, 3868, CST), anti-DHODH (1:2000, 14877-1-AP, Proteintech), anti-AIFM2 (1:1000, sc-377120, Santa Cruz), anti-MBOAT1 (1:1,000, 25615-1-AP, Proteintech), anti-MBOAT2 (1:1,000, AP17786c, Abcepta), anti-K63-linkage Specific Polyubiquitin (1:1000, A18164, ABclonal), anti-Vinculin (1:1,000, V9131, Sigma) and anti-β-Actin (1:1,000, sc-47778, Santa Cruz). The following secondary antibodies were used: anti-rabbit IgG-HRP (1:5,000, sc-2357, Santa Cruz) and anti-mouse IgGκ-HRP (1:5,000, sc-516102, Santa Cruz).

**Metabolomic analysis and isotope tracing**

**Untargeted metabolomic analysis.** ALDOA-depleted and control 786-O cells were seeded in 15 cm culture dishes and cultured until reaching 90% confluency. Cells were washed once with cold DPBS and scraped off using 1 mL cold methanol/acetonitrile (1:1, v/v) to extract metabolites. The mixture was transferred to a centrifuge tube and spun at 14,000 g for 20 min (4 °C). The supernatant was collected and dried in a vacuum centrifuge. The pellets were dissolved in KOH, and protein concentrations were measured by Pierce BCA assay (Thermo Fisher) for normalization. Prior to LC–MS, dried samples were re-dissolved in 100 µL acetonitrile/water (1:1, v/v), centrifuged at 14,000 g for 15 min (4 °C), and the supernatant used for subsequent LC–MS analysis.

LC–MS/MS analysis was performed on a Vanquish UHPLC (Thermo) coupled to an Orbitrap Exploris 480 mass spectrometer (Thermo Fisher). For HILIC separation, a 2.1 mm × 100 mm ACQUITY UPLC BEH Amide column (1.7 µm, Waters) was used. In both positive and negative ESI modes, mobile phase A was 25 mM ammonium acetate + 25 mM ammonium hydroxide in water, and mobile phase B was acetonitrile. The gradient was: 0.5 min, 95% B; 7.0 min, 65% B; 8.0 min, 40% B; 8.1 min, 95% B (hold for 2.9 min). The ESI source settings were: Gas1 = 50, Gas2 = 2, source temperature 350 °C, ISVF +3,500 V / –2,800 V. In MS-only mode, m/z 70–1,200 Da was scanned at resolution 60,000 with 100 ms accumulation time. In auto MS/MS mode, the same m/z range and resolution (60,000) and accumulation time (100 ms) were used, with an exclusion period of 4 s.

Raw data were converted to mzXML by ProteoWizard MSConvert and processed with XCMS (centWave m/z tolerance = 10 ppm, peakwidth 10–60 s, prefilter = c(10,100)). Peak grouping used bw = 5, mzwid = 0.025, minfrac = 0.5. CAMERA was used to annotate isotopes/adducts. Only features with >50% nonzero values in at least one group were retained. Metabolite IDs were assigned by matching precise m/z (<10 ppm) and MS/MS spectra to an in-house standard library. After sum normalization, data were analyzed in R using the ropls package for PCA and OPLS-DA. A 7-fold cross-validation and permutation test were used to assess model robustness. Variables with VIP > 1 and p < 0.05 (Student’s t-test) were considered significantly altered. Pearson correlation was used to evaluate inter-metabolite relationships.

**Isotope labeling and isotope tracing analysis.** For glucose tracing analysis, RPMI1640 medium without glucose (Gibco, 11879020) was supplemented with either glucose or U-^13^C-glucose (Cambridge Isotope Laboratories, CLM-1396), along with 10% dialyzed FBS (Gibco, 30067334). ALDOA-depleted and control 786-O/MCF10A cells were seeded in 6 cm dishes, and transferred into isotopic labeling medium or control medium. After 0.5 h of labeling, cells were washed once with ice-cold DPBS, quenched with pre-chilled methanol/water (80:20, v/v), and held at –80 °C for 2 h. Cells were scraped, and extracts were centrifuged at 14,000 g for 20 min (4 °C). The supernatant was used for metabolite analysis and pellets were dissolved in KOH for BCA quantification for normalization.

LC–MS/MS tracing was conducted using a Vanquish™ Flex UPLC system coupled to a TSQ Quantiva Ultra triple-quadrupole mass spectrometer (Thermo Fisher, CA), equipped with a heated ESI (HESI) source. Extracts were separated by a Synergi Hydro-RP column (2.0×100mm, 2.5 μm, phenomenex). A binary solvent system was applied, in which mobile phase A comprised 10 mM tributylamine adjusted with 15 mM acetic acid in water, and mobile phase B was 100% methanol. A linear 25-minute gradient was used, progressing from 5% B to 90% B. Data were collected in SRM mode with positive/negative ion switching. The resolution for Q1 and Q3 are both 0.7 FWHM. The source voltage was 3500v for positive and 2500v for negative ion mode. The source parameters are as follows: capillary temperature: 350 °C; heater temperature: 300 °C; sheath gas flow rate: 35; auxiliary gas flow rate: 10. Tracefinder 3.2 (Thermo, USA) was applied for metabolite identification and peak integration. Data analysis and natural isotope abundance correction were performed with MassHunter Profinder software (Agilent). Relative levels of M+n isotopologues were quantified from corrected peak areas and normalized to total biomass.

**Targeted LC–MS/MS analysis of F1,6BP.** ALDOA-depleted and control 786-O cells, as well as ALDOA-depleted 786-O cells reconstituted with WT or D34S mutant ALDOA, were cultured in 10-cm dishes until reaching 90% confluency. Next, samples were collected and prepared for LC-MS/MS analysis as described in untargeted metabolomic analysis, using pre-chilled methanol/water (80:20, v/v) as extraction solvent. The BCA assay was conducted to normalize cell numbers between different samples.

Targeted F1,6BP analysis was performed on a TSQ Quantiva triple-quadrupole mass spectrometer (Thermo Fisher) with ESI source, using C18 reverse-phase chromatography. Mobile phase A = 10 mM tributylamine + 15 mM acetate in water, B = 100% methanol. The gradient was: 0 min, 5% B; 25 min, 90% B; followed by re-equilibration at 5% B for 5 min. Flow rate 0.3 mL/min, column temp 35 °C. MS acquisition was conducted in negative-polarity selective reaction monitoring (SRM) mode. Q1 and Q3 resolution were 0.7 FWHM. Source settings: spray voltage 3,000 V; capillary 320 °C; heater 300 °C; sheath gas 35; auxiliary gas 10. The source voltage was 3,500 V (positive) and 2,500 V (negative). Authentic standards of F1,6BP were used for calibration. The SRM transition for F1,6BP is 338.95 → 97.1 with a retention time of ~13.9 min. Quantification and metabolite identification were done using TraceFinder with a home-built standard library.

**Quantitative lipidomic profiling and data analyses**

**Sample preparation and lipid extraction.** For sample preparation, ALDOA-depleted and control 786-O, BT-474 and MCF10A cells were trypsinized and counted within a short period of time. Equal numbers of cells were then collected for lipidomic analysis. Subsequently, lipids were extracted according to the methyl tert-butyl ether (MTBE) method. Briefly, samples were mixed with 200 μl methanol (Themo Fisher), and then supplemented with 10 μl internal lipid standards and 800 μl methyl tert-butyl ether (MTBE). For the internal lipid standards, SPLASH® LIPIDOMIX® Internal Standard (330707-1EA) and C15-Ceramide-d7 (d18:1-d7/15:0) (Avanti, 860681P-1mg) were used. The mixture was adequately vortexed, sonicated for 20 min at 4 °C and then kept for 30 min at room temperature. Subsequently, 200 μl of MS-grade water was added, and the mixture was vortexed and centrifuged at 14,000 rpm for 15min at 4 °C. The upper organic solvent layer was collected and dried under nitrogen. For LC-MS analysis, the samples were re-dissolved in 200 μl of IPA/ACN (9:1, v/v) solvent and centrifuged at 14,000 rpm at 4 °C for 15 min, and then the supernatant was injected.

**LC-MS/MS method.** Lipidomic analysis was performed on a UHPLC system (LC-30AD, Shimadzu) coupled with QTRAP 6500+ mass spectrometer (Sciex). Separation was achieved using either a HILIC column (Phenomenex Luna NH₂, 2.0 × 100 mm, 3 µm) or a C18 column (Phenomenex Kinetex C18, 2.1 × 100 mm, 2.6 µm). For C18 separation, the column temperature was set at 45 °C. Mobile phase A: 70% acetonitrile + 30% H_2_O + 5 mM ammonium acetate, mobile phase B: IPA solution. A gradient (20% B at 0 min, 60% B at 5 min, 100% B at 13 min, 20% B at 13.1-17 min) was then initiated at a flow rate of 0.35 ml/min. The sample was placed at 10 °C during the whole analysis process. For HILIC (Amino) separation,, the column temperature was set at 40 °C. Mobile phase A: 2mM ammonium acetate + 50% methanol + 50% acetonitrile, and mobile phase B: 2 mM ammonium acetate + 50% acetonitrile + 50% water. A gradient (3% B at 0-3 min, from 3% to 100% B at 3-13 min, 100% B at 13-17 min, 3% B at 17.1-22 min) was then initiated at a flow rate of 400 μl/min. The QTRAP 6500+ operated in positive/negative electrospray ionization (ESI) switch mode. The ESI positive source conditions were as follows: Source temperature: 400 °C; Ion Source Gas1 (GS1): 50 Ion Source Gas2 (GS2): 55; Curtain Gas (CUR): 35; IonSpray Voltage (IS): +3000 V; The ESI negative source conditions were as follows: Source temperature: 400 °C; Ion Source Gas1 (GS1): 50; Ion Source Gas2 (GS2): 55; Curtain gas (CUR): 35; IonSpray Voltage (IS): -2500 V. MRM method was used for mass spectrometry quantitative data acquisition. Polled quality control (QC) samples were set in the sample queue to evaluate the stability and repeatability of the system.

**Data processing.** Raw data were processed using Sciex OS software. The QCs were processed together with the biological samples. Metabolites in QCs with coefficient of variation (CV) less than 30% were denoted as reproducible measurements. Lipid identification was based on matching to authentic standards, and lipid composition and differential abundance analyses were subsequently performed.

**Quantitative proteomic profiling and data analysis**

**Sample preparation.** ALDOA-depleted and control 786-O cells were seeded in 15 cm culture dishes and cultured until reaching 90% confluency. Cells were washed once with cold DPBS and scraped off the plate using SDT buffer (4% SDS, 100 mM Tris-HCl, pH 7.6). Lysates were further sonicated and boiled for 15 min. After centrifuged at 14,000 g for 40 min, the supernatant was quantified with the BCA protein assay kit. 15 μg of protein for each sample were mixed with 5 × loading buffer respectively and boiled for 5 min. The proteins were separated on 4-20% SDS-PAGE gel. Protein bands were visualized by Coomassie Blue R-250 staining.

**Filter-aided sample preparation (FASP digestion).** DTT was added to each sample to a final concentration of 40 mM, and the mixtures were shaken at 600 rpm for 1.5 h at 37°C. fter cooling to room temperature, IAA was added to a final concentration of 20 mM into the mixture to alkylate cysteine residues, and samples were incubated in the dark for 30 min. Samples were then transferred to the filters (Microcon units, 10 kDa). Filters were washed with 100 μl UA buffer three times and twice with 100 μl 25 mM NH_4_HCO_3_ . Proteins were digested with trypsin at a 1:50 (enzyme:protein, w/w) ratio and incubated at 37 °C overnight (15–18 h). and then the resulting peptides were collected as a filtrate. The resulting peptides were collected as filtrates, desalted on C18 cartridges (Empore™ SPE Cartridges MCX, 30 µm; Waters), concentrated by vacuum centrifugation, and reconstituted in 40 µl 0.1% formic acid (v/v). Peptide concentration was estimated by UV absorbance at 280 nm. For data-independent acquisition (DIA) analysis, iRT (indexed retention time) calibration peptides were spiked into each sample.

**Mass spectrometry assay for data independent acquisition (DIA).** Peptides were analyzed using an Orbitrap™ Astral™ mass spectrometer (Thermo Fisher Scientific) coupled to a Vanquish Neo UHPLC system (Thermo Fisher Scientific) operating in data-independent acquisition (DIA) mode. Precursor ions were scanned from m/z 380–980, with an MS1 resolution of 240,000 at m/z 200, normalized AGC target 500%, and maximum injection time (IT) 5 ms. For MS2, 299 DIA windows were acquired with isolation window 2 m/z, HCD collision energy 25 eV, normalized AGC target 500%, and maximum IT 3 ms.

**Mass spectrometry data analysis.** DIA data was analyzed with DIA-NN v.1.8.1. Parameters were set as follows: enzyme: trypsin; maximum missed cleavages: 1; fixed modification: carbamidomethyl (C); variable modifications: oxidation (M) and acetylation (protein N-terminus). Protein identification was filtered at false discovery rate (FDR) ≤ 1%, corresponding to 99% confidence.

**Ubiquitination site mapping of MBOAT2 by immunoprecipitation and LC–MS/MS**

**Sample preparation.** HEK293T cells were transiently transfected with a pcDNA3.1-MBOAT2-cFlag expression construct for 48 h. Where indicated to stabilize autophagy-dependent ubiquitin conjugates, cells were treated with bafilomycin A1 (100 nM) for 4 h prior to collection. Cells were lysed on ice for 30 min in Pierce RIPA buffer (Thermo Fisher) supplemented with 10 mM N-ethylmaleimide (NEM), 50 µM PR-619, and a protease inhibitor cocktail (Roche). Lysates were clarified by centrifugation at 17,000 × g for 10 min at 4 °C. Cleared lysates were incubated with DYKDDDDK tag Nanoselector Magnetic beads (AlpVHHs) at 4 °C for 1 h with gentle rotation. Beads were washed four times with ice-cold wash buffer (10 mM Tris-HCl, pH 7.5; 150 mM NaCl; 0.05% NP-40; 0.5 mM EDTA). Bound proteins were eluted by boiling in 2× Laemmli sample buffer (BioRad). Eluates were separated by SDS–PAGE, and Coomassie-stained bands above the expected molecular weight of MBOAT2 were excised. Proteins were reduced with 5 mM DTT (30 min, 56 °C) and alkylated with 11 mM iodoacetamide (30 min, room temperature, dark). In-gel digestion was performed overnight at 37 °C with sequencing-grade modified trypsin in 50 mM ammonium bicarbonate (ABC). Peptides were extracted twice with 1% TFA in 50% acetonitrile (1 h each), pooled, concentrated in a SpeedVac, resuspended in 20 µL 0.1% TFA, and clarified by centrifugation (20,000 × g, 15 min, 4 °C).

**LC–MS/MS and data processing.** Peptides were analyzed on a Thermo Dionex Ultimate 3000 nano-HPLC coupled to a Thermo Orbitrap Fusion Lumos mass spectrometer. Peptides were loaded onto a home-packed C18 column (75 µm ID × 150 mm, 5 µm, 300 Å) and separated at 0.3 µl/min using a 40 min gradient of mobile phase A (0.1% formic acid in water) and mobile phase B (0.1% formic acid in acetonitrile). Data-dependent acquisition (DDA) was performed in Xcalibur 4.0.27.10, with full MS scans acquired in the Orbitrap (m/z 300–1500, 120,000 resolution), followed by top-speed MS/MS for 3 s using HCD (NCE 40%) and ion-trap detection. Raw files were searched against the human UniProt reference proteome using Proteome Discoverer v1.4 (Sequest HT). Search parameters: Enzyme: trypsin (≤ 2 missed cleavages); precursor tolerance: 10 ppm; fragment tolerance: 0.8 Da; Fixed modification: carbamidomethyl-Cys; Variable modifications: oxidation-Met; Lys-ε-diglycine (K-ε-GG, +114.04293 Da) on Lys for substrate-site mapping and on ubiquitin peptides for chain-type inference. Peptide-spectrum matches (PSMs) were filtered to 1% FDR (target–decoy), and key spectra were manually inspected. MBOAT2 ubiquitination sites were called when high-confidence K-ε-GG peptides detected in MBOAT2 IP samples. Ubiquitin chain types were inferred from K-ε-GG–modified ubiquitin peptides.

**Immunoprecipitation of MBOAT2 and detection of K63-linked ubiquitination**

To obtain sufficient protein for immunoprecipitation-based ubiquitination analyses, 786-O cells stably overexpressing MBOAT2 (via lentiviral transduction) were used. For each immunoprecipitation (IP), cells from one confluent 15-cm dish were collected. Where indicated, cells were treated with 100 nM Bafilomycin A1 (BafA1) for 4 h prior to harvesting to inhibit autophagic degradation and stabilize K63-linked ubiquitinated proteins. Cells were washed twice with ice-cold PBS and lysed on ice for 30 min in Pierce RIPA buffer (Thermo Fisher) supplemented with 10 mM N-ethylmaleimide (NEM;Sigma), 50 µM PR-619 (MedChemExpress), and protease inhibitor cocktail (Roche) to prevent deubiquitination and proteolysis. Lysates were clarified by centrifugation at 17,000 × g for 10 min at 4 °C, and protein concentrations were determined using the Pierce BCA protein assay kit (Thermo Fisher). For immunoprecipitation, 5 mg total protein was incubated with 4 µg anti-MBOAT2 antibody or 4 µg normal rabbit IgG (CST, 2729) overnight at 4 °C with gentle rotation. Subsequently, 30 µL of pre-washed Rabbit lgG Nanoselector Magnetic beads (AlpVHHs) were added and incubated for an additional 1 h at 4 °C. The beads were washed four times with ice-cold wash buffer (10 mM Tris-HCl, pH 7.5; 150 mM NaCl; 0.05% NP-40; 0.5 mM EDTA), each wash for 5 min at 4 °C with gentle rotation. Bound proteins were eluted by boiling the beads in 2× Laemmli sample buffer (Bio-Rad) containing β-mercaptoethanol for 5 min. Eluted immunocomplexes, along with 5–10% input lysates, were subjected to SDS–PAGE and immunoblotting. Membranes were probed with antibodies against K63-linked polyubiquitin and MBOAT2; Vinculin or β-actin served as loading controls for input samples. The degree of K63-linked ubiquitination was quantified as the ratio of K63-Ub (IP) to MBOAT2 (IP) within the same lane using ImageJ v.2.16.

**Animal studies**

All mice were maintained under specific pathogen-free (SPF) conditions, and used in accordance with protocols approved by the Institutional Animal Care and Use Committees (IACUC) of Tsinghua University for animal welfare. All of the animals used were matched for age and sex and randomly allocated to experimental groups. The study is compliant with all of the relevant ethical regulations regarding animal research. For the tumor xenograft model, female 4-week-old athymic nude mice were purchased from Charles River (Beijing Vital River Laboratory Animal Technology). The mice were fed with sterile pellet food and water ad libitum, and were kept under a 12/12 h light/dark cycle at 22-26 °C.

**Subcutaneous tumor model and treatment.** For the ALDOA-depleted BT-474 subcutaneous tumor model, 4-week-old female BALB/c athymic nude mice were implanted with 0.72-mg, 60-day release 17 β-estradiol pellets (Innovative Research of America). The following day, BT-474 cells expressing shCtrl or shALDOA were collected by trypsinization and counted. Then, 5 × 10^6^ BT-474 cells expressing shCtrl or shALDOA in 100 μl mixture of DPBS and Matrigel matrix (BD Bioscience) (volume ratio 1:1) were injected separately on the left and right flank of BARB/c athymic nude mice. When the tumor volume reached about 100 mm^3^ (length × width^2^ × 1/2), we started to treat mice with IKE (40 mg/kg, 5% DMSO-dissolved IKE stock solution + 40% PEG300 + 5% Tween-80 + 50% ddH_2_O, once every day), IKE with Lip-1 (IKE 40 mg/kg, Lip-1 10 mg/kg, 5% DMSO-dissolved IKE and Lip-1 stock solution + 40% PEG300 + 5% Tween-80 + 50% ddH_2_O, once every day), or vehicle control (5% DMSO + 40% PEG300 + 5% Tween-80 + 50% ddH_2_O, once every day) intraperitoneally, and tumor volume was measured every 2 days until the end point.

For the ALDOA inhibitor LYG-202 treated BT-474 subcutaneous tumor model, 4-week-old female BALB/c athymic nude mice were implanted with 0.72-mg, 60-d release 17β-estradiol pellets. The following day, 5 × 10^6^ BT-474 cells in 100 μl equal volume of DPBS and Matrigel matrix were injected on the right flank of athymic nude mice. When the tumor volume reached about 100-200 mm^3^, mice were treated with LYG-202 (50 mg/kg, 10% ethanol-dissolved LYG-202 stock solution + 30% PEG400 + 60% Phosal 50 PG, once every other day), IKE (80 mg/kg, 10% ethanol-dissolved IKE stock solution + 30% PEG400 + 60% Phosal 50 PG, once every other day), LYG-202 + IKE (50 mg/kg LYG-202 and 80 mg/kg IKE, 10% ethanol-dissolved LYG-202 and IKE mixed solution + 30% PEG400 + 60% Phosal 50 PG, once every other day), LYG-202 + IKE + Lip-1 (50 mg/kg LYG-202, 80 mg/kg IKE and 10 mg/kg Lip-1, 10% ethanol-dissolved mix solution + 30% PEG400 + 60% Phosal 50 PG, once every other day), or vehicle control (10% ethanol + 30% PEG400 + 60% Phosal 50 PG, once every other day) intraperitoneally. Tumor volume was measured every 2 days until the end point. The maximal tumor volume was 2,000 mm^3^ and this limit was not exceeded in any of the experiments.

**Histology and** **immunohistochemistry staining**

Tissues were fixed in 4% paraformaldehyde, dehydrated in a graded ethanol series and xylene, and then embedded in paraffin. Embedded samples were sectioned at a thickness of 3-5 μm for H&E staining or immunohistochemistry staining according to standard protocol. For immunohistochemistry, the following antibodies were used: anti-4HNE (1:100, ab46545, Abcam), Aldolase A (1:100, sc-390733, Santa Cruz), Ki-67 (1:50, 550609, BD Bioscience) and cleaved caspase-3 (1:400, 9661, CST). In brief, paraffin-embedded samples were cut into 5 μM sections and deparaffinized using xylene and hydrated. Subsequently, deparaffinized sections were heated for antigen retrieval in a microwave oven submersed in 0.01 M citrate buffer pH 6.0. After cooling, sections were blocked overnight at 4 °C with 5% FBS and 0.05% Tween-20 in TBS (pH 7.4). The next day, after incubation with 0.3% H_2_O_2_ in methanol for 20 min, the sections were incubated with the following secondary antibodies for 60 min: biotinylated goat anti-rabbit IgG (1:100, A0279, Beyotime) for anti-4-HNE and anti-cleaved caspase-3 antibodies; biotinylated goat anti-mouse-IgG (1:100, A0288, Beyotime) for anti-ALDOA and anti-Ki-67 antibodies, and then incubated with streptavidin–biotin peroxidase complex (Beyotime, P0603). The sections were visualized with DAB solution (Beyotime, P0203) and counterstaining with Mayer’s Hematoxylin. Images were acquired using 3DHISTECH Pannoramic whole-slide scanner. IHC results were quantified and statistically analyzed using ImageJ v.2.16 and GraphPad Prism 9 software.

**Statistical analysis**

Statistical analyses were conducted using GraphPad Prism 9 software. Detailed statistical information for each experiment is provided in the corresponding figure legends. The number of independent biological or technical replicates, as well as the number of independent experiments performed, is reported in the corresponding figure legends. Values are presented as mean ± s.d. for cell culture experiments and mean ± s.e.m. for animal experiments. Statistical significance for pairwise comparisons was calculated using the two-tailed unpaired Student’s t-test. One-way ANOVA or two-way ANOVA was used for comparing multiple experimental groups. Results were considered statistically significant at P < 0.05.
